# Stable Ultrabroad‐Absorbing Radical Achieves Efficient NIR‐II Photothermal Conversion via Facile Synthesis

**DOI:** 10.1002/advs.202513587

**Published:** 2025-09-03

**Authors:** Haozhe Zhang, Yuhang Yang, Jiaxing Huang, Luotai Chen, Yongyan Cui, Zhiyuan Lu, Shishi Shen, Zixi Liu, Hongwei Song, Zhuoran Kuang, Qianxi Dang, Yuan Li

**Affiliations:** ^1^ State Key Laboratory of Luminescent Materials and Devices Institute of Polymer Optoelectronic Materials and Devices School of Materials Science and Engineering South China University of Technology Guangzhou Guangdong 510640 P. R. China; ^2^ State Key Laboratory of Information Photonic and Optical Communications and School of Science Beijing University of Posts and Telecommunications Beijing 100876 P. R. China; ^3^ Department of Plastic and Reconstructive Surgery Peking University Shenzhen Hospital Shenzhen Guangdong 518036 P. R. China; ^4^ Guangdong Provisional Key Laboratory of Functional Oxide Materials and Devices Southern University of Science and Technology Shenzhen Guangdong 518055 P. R. China

**Keywords:** donor–acceptor, electron acceptor, open‐shell, photothermal conversion, radical

## Abstract

Inspired by the electron‐withdrawing ability of nitroxide radicals, a novel open‐shell material, EDOT‐TPAO_4_ is reported, synthesized via one‐step demethylation and oxidation of its closed‐shell precursor, EDOT‐TPAOMe_4_. Time‐dependent density functional theory calculations confirm an acceptor–donor–acceptor configuration of EDOT‐TPAO_4_ where radical termini act as electron acceptors. This structural transformation narrows the optical bandgap from 2.74 eV of EDOT‐TPAOMe_4_ to 1.26 eV of EDOT‐TPAO_4_ and endows EDOT‐TPAO_4_ high electrical conductivity of 0.02 S cm^−1^ and unexpected electrochemical stability in air. The reduced bandgap enables ultrabroad absorption in powder (300–2500 nm), driving exceptional photothermal conversion. Under 1064 nm laser irradiation (0.9 W cm^−2^), EDOT‐TPAO_4_ heats rapidly to 290 °C within 60 s, outperforming most reported pure organic photothermal materials. Femtosecond spectroscopy confirms ultrafast excited‐state nonradiative quenching in picosecond timescale, underlying the efficient photothermal conversion. Leveraging both its optical properties and thermal responsiveness, EDOT‐TPAO_4_ achieves outstanding solar‐driven water evaporation of 1.433 kg m^−2^ h^−1^. This study not only presents one of the most efficient and readily synthesized organic photothermal materials to date, but also establishes a new molecular design strategy that expands the functional landscape of next‐generation radical‐based electron acceptor building blocks and open‐shell radical semiconductors for electronics, challenging conventional closed‐shell strategies in organic electronics.

## Graphical abstract

A new and stable open‐shell acceptor–donor–acceptor type radical EDOT‐TPAO_4_ with electronic conductivity of 0.02 S cm^−1^ is prepared by one‐step with high yield and exhibited unexpected photothermal conversion as one of the best pure organic materials.

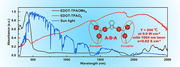

## 1 Introduction

Photothermal conversion materials have become crucial elements in sustainable energy technologies and advanced biomedical treatments,^[^ ^1^, ^2^, ^3^, ^4^, ^5^, ^6^, ^7^, ^8^ ^]^ significantly advancing fields such as solar‐driven water evaporation, photothermal therapy, and thermoelectric generation systems.^[^ ^9^, ^10^, ^11^, ^12^, ^13^, ^14^, ^15^, ^16^, ^17^ ^]^ Organic small‐molecule materials represent promising alternatives due to their structural tunability, straightforward synthetic accessibility, and compatibility with solution processing techniques.^[^ ^18^, ^19^, ^20^, ^21^ ^]^ However, organic photothermal materials typically encounter significant challenges, including narrow absorption spectra, limited photothermal stability, and inefficient non‐radiative decay pathways. Therefore, it is important to design new organic small‐molecules capable of broad‐spectrum NIR absorption, robust photothermal stability, and efficient non‐radiative thermal conversion.

**Figure 1:**
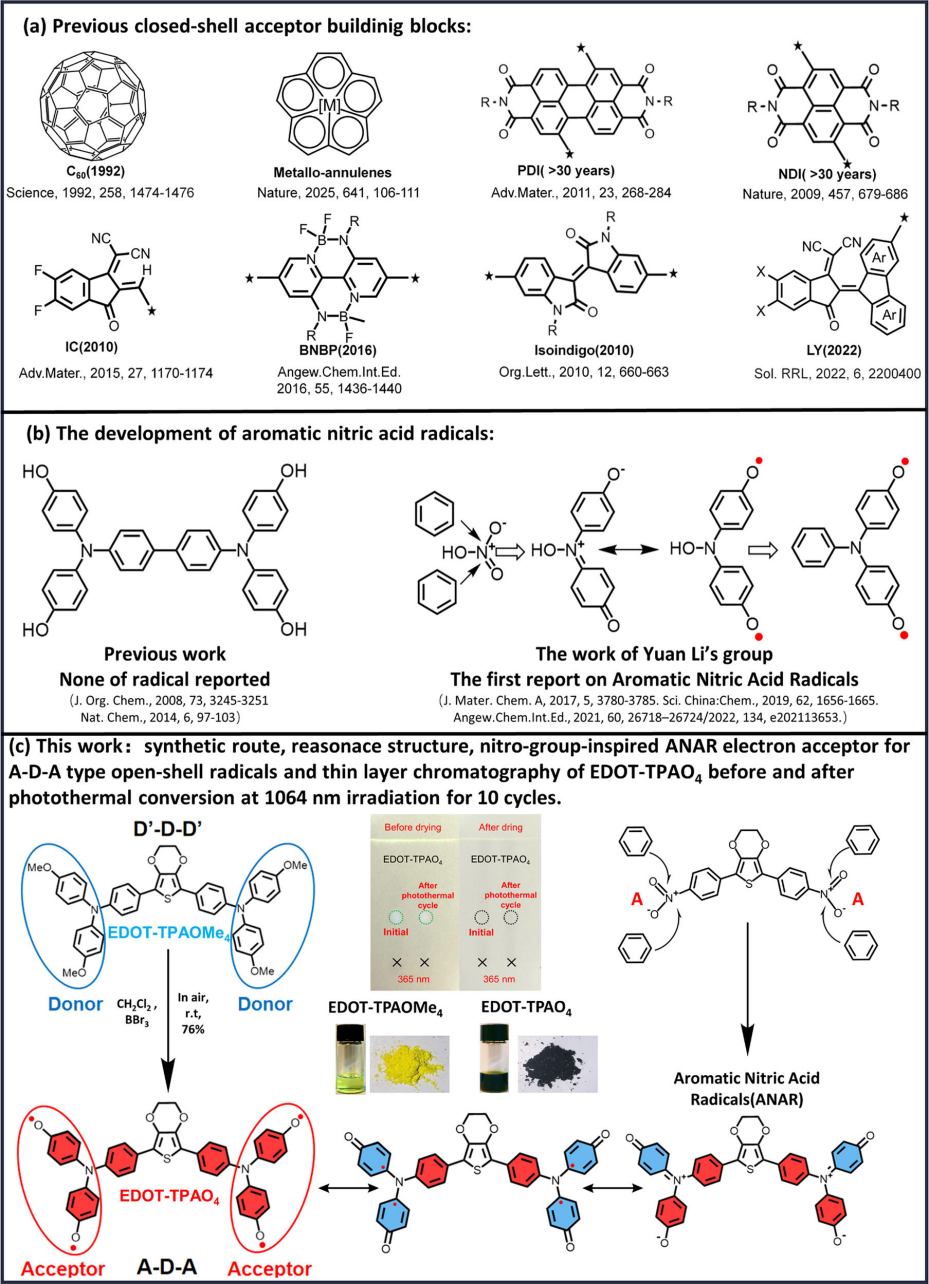
a) The previous work on acceptor research.^[^ ^25^, ^32^, ^33^, ^34^, ^35^, ^36^, ^63^, ^64^ ^]^ b) The previous work^[^ ^46^, ^47^ ^]^ and development of aromatic nitric acid radicals work in Group of Li^[^ ^41^, ^45^, ^48^ ^]^). c) This work on open‐shell radicals as electron acceptors to construct A‐D‐A type low band photothermal conversion materials, the thin layer chromatography results of the EDOT‐TPAO_4_ sample before and after 1064 nm photothermal cycles.

Generally, D‐A structures can modulate the absorption and emission properties of small molecules.^[^ ^22^, ^23^ ^]^ The acceptor–donor–acceptor (A‐D‐A) molecular framework, initially successful in organic photovoltaics,^[^ ^24^ ^]^ has attracted considerable attention owing to its extensive π‐conjugation, and optimal frontier orbital alignment.^[^ ^1^, ^25^, ^26^, ^27^, ^28^, ^29^, ^30^ ^]^ Representative A‐D‐A acceptors, such as ITIC,^[^ ^25^ ^]^ Y6,^[^ ^31^ ^]^ and their derivatives, have greatly advanced photovoltaic efficiencies. **Figure** **1**a delineates the previously reported organic acceptor units. As an early‐discovered acceptor molecule, C_60_ has exerted a profound influence on subsequent acceptor development.^[^ ^32^ ^]^ Based on the design of stable electron‐deficient backbones—such as naphthalene diimide (NDI),^[^ ^33^ ^]^ perylene diimide (PDI),^[^ ^34^ ^]^ isoindigo,^[^ ^35^ ^]^ B←N bridged bipyridine (BNBP),^[^ ^36^ ^]^ benzothiadiazole,^[^ ^37^ ^]^ and boron‐difluoride complexes (eg. BODIPY)^[^ ^38^, ^39^ ^]^ —the system of organic electron acceptor materials has continuously evolved. Especially, the latter two have demonstrated superior performance in the photothermal field. In 2025, the planar metallo‐annulenes acceptor developed by Xia's group elevated this field to unprecedented heights.^[^ ^40^ ^]^ However, most of these systems still rely almost exclusively on closed‐shell acceptors, in contrast to open‐shell systems that exhibit longer‐wavelength absorption and enhanced photothermal performance.^[^ ^41^, ^42^ ^]^ Moreover, photothermal materials containing these closed‐shell receptors typically undergo multi‐step synthesis processes and exhibit limited thermoelasticity under strong NIR irradiation.^[^ ^43^, ^44^ ^]^ Despite their structural diversity, existing A‐D‐A type organic photothermal materials rarely leverage the open‐shell properties—a potential avenue for enhancing photothermal performance—to regulate the absorption and emission properties of molecules.

In our previous work, we first reported the aromatic nitric acid radicals (ANAR), which greatly enriched our understanding of phenolamines (Figure 1b).^[^ ^45^ ^]^ Compared with previously published phenoxide radicals,^[^ ^46^, ^47^ ^]^ ANAR do not require bulky steric protection and can maintain radical stability via extensive resonance delocalization.^[^ ^41^, ^42^, ^45^, ^48^ ^]^ Building upon this groundbreaking discovery, we have consistently pursued the development of a series of ANAR materials.^[^ ^41^, ^48^, ^49^, ^50^, ^51^, ^52^, ^53^, ^54^, ^55^ ^]^ Open‐shell radicals—particularly aromatic nitroxide radicals—offer high intrinsic spin polarization, and excellent redox reversibility,^[^ ^45^, ^56^, ^57^ ^]^ which collectively promote non‐radiative decay channels.^[^ ^41^, ^42^, ^58^, ^59^, ^60^, ^61^, ^62^ ^]^ While radicals have been extensively studied in the context of spintronic devices and photophysical modulation,^[^ ^59^ ^]^ their role as electron acceptors has seldom been explored, let alone systematically employed in A‐D‐A organic frameworks. This work aims to challenge this prevailing assumption by proposing a new design strategy that incorporates open‐shell radical acceptor units into conjugated systems, thereby fundamentally redefining the design space of organic photothermal materials.

Herein, we developed a novel open‐shell A‐D‐A molecule, EDOT‐TPAO_4_, by introducing two ANAR acceptor building blocks symmetrically onto the electron‐donating EDOT donor core through a single‐step step demethylation of the commercially available and low cost methoxy precursor EDOT‐TPAOMe_4_ with a high yield of 76% (Figure 1c). Moreover, we confirm that EDOT‐TPAO_4_ adopts an A‐D‐A configuration through time‐dependent density functional theory calculations, while its methoxy precursor exhibits a D’‐D‐D’ configuration. This provides theoretical support for our finding that the radicals on both sides of the molecule act as electron acceptors. This radical‐enabled architecture demonstrates a narrow optical bandgap (1.26 eV) and stable electrochemical property (The curve barely fluctuates after twenty cycles). Unlike traditional closed‐shell acceptors that require complex synthesis to reach narrow bandgaps and long‐wavelength absorption (Figure 1a), EDOT‐TPAO_4_ achieves broadband absorption from 300 to 2500 nm with remarkable synthetic simplicity and processability. Interestingly, under 1064 nm laser irradiation (0.9 W cm^−2^), EDOT‐TPAO_4_ rapidly heats to 290 °C within 60 s, outperforming most reported organic photothermal materials. Femtosecond spectroscopy analysis reveals that the material demonstrates a rapid intersystem crossing in the aggregated state, leading to prompt triplet‐state quenching and thereby achieving efficient photothermal conversion, which is fully consistent with our experimental results. It further demonstrates excellent thermal/photo stability, reusability, and solution processability, enabling practical scalability. In functional applications, it delivers an impressive solar‐driven water evaporation rate of 1.433 kg m^−2^ h^−1^ and high energy conversion efficiency, while also supporting low‐threshold laser ignition, thereby highlighting its versatility across photothermal platforms.

By integrating open‐shell radical acceptors into an A‐D‐A molecular framework, this study introduces a previously unexplored electronic configuration into photothermal material design. The presented strategy not only offers a scalable and efficient pathway toward NIR‐II‐active organic photothermal agents, but also redefines the electron‐acceptor paradigm in conjugated molecular systems.

## 2 Results and Discussion

### 2.1 Synthesis and Characterization of EDOT‐TPAO_4_ Radical

The target open‐shell radical compound EDOT‐TPAO_4_ was extremely readily synthesized via the simple and well‐known demethylation reaction of the commercially available raw material EDOT‐TPAOMe_4_ using boron tribromide in DCM at room temperature. Then, the crude product was then very carefully purified by silica gel column chromatography (ethanol:ethyl acetate, 1:1) to afford a pure black powder with a high yield of 76%, which is similar with our previous work (Figure S1, Supporting Information). The ^1^H‐NMR spectrum of the product EDOT‐TPAO_4_ (Figure S2, Supporting Information) was conducted to confirm the purity of our radical compound.^[^ ^42^, ^63^, ^65^ ^]^ The signal in ^1^H‐NMR spectrum is attributed to hydrolysis of the majority of radical with trace water in DMSO‐*d*_6_, forming the hydroxylated by‐product EDOT‐TPAOH_4_, and the residual radicals do not significantly compromise spectral quality, so the signals is well consistent with the assignment EDOT‐TPAOH_4_.^[^ ^42^, ^63^, ^65^ ^]^ None of the polymer or by‐products were detected from the clean ^1^H‐NMR spectrum of EDOT‐TPAO_4_ and the thin‐layer chromatography results in Figure 1c definitely further confirmed the high impurity of the pure radical character of EDOT‐TPAO_4_. The matrix‐assisted laser desorption/ionization time of flight mass spectrometry analysis (Figure S3, Supporting Information) revealed a prominent peak at m/z 692.12, corresponding to the [M+4H]^+^ ion of EDOT‐TPAOH_4_, which matched the theoretical molecular weight of EDOT‐TPAOH_4_, due to the radical cation species,^[^ ^42^, ^63^, ^65^ ^]^ confirming successful synthesis and high purity of EDOT‐TPAO_4_.

### 2.2 UV–Vis–NIR Absorption and Photophysical Property

**Figure 2:**
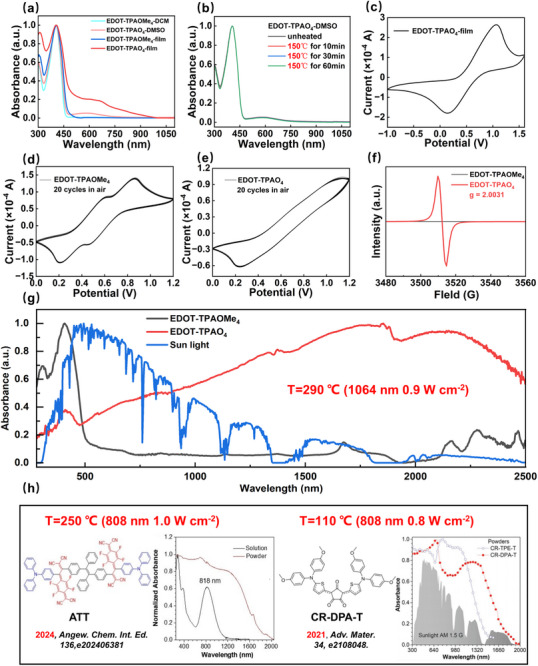
a) UV–vis–NIR absorption spectra in DMSO solvent and film compared with precursors. b) UV–vis–NIR absorption spectra in DMSO after heating. c) 20 cycles CV curve of EDOT‐TPAMe_4_ in DCM. d) 20 cycles CV curve of EDOT‐TPAO_4_ in THF. e) CV curve of EDOT‐TPAO_4_ in film. f) Electron paramagnetic spectra of powder samples. g) The normalized absorption spectra in powder state compared with precursors. h) Comparison of previous photothermal material powder absorption spectra.^[^ ^67^, ^68^ ^]^

The optical properties of the two compounds were studied by UV–vis–NIR absorption spectra (**Figure** **2**a). Interestingly, EDOT‐TPAO_4_ exhibited significantly different optical properties from those of EDOT‐TPAOMe_4_ (Figure 2a), which are in good agreement with our previous work on ANAR structure formation of the open‐shell EDOT‐TPAO_4_.^[^ ^42^, ^48^, ^66^ ^]^ EDOT‐TPAO_4_ and EDOT‐TPAOMe_4_ have similar absorption in the short wavelength range in solution, however, EDOT‐TPAO_4_ exhibited a new broad and relatively weak absorption band between 450 and 760 nm, which is the typical absorption behavior of the ANARs.^[^ ^42^, ^45^, ^48^, ^66^ ^]^ The absorption edge of the EDOT‐TPAO_4_ film reached ≈ 985 nm, which illustrates its lowered bandgap due to its A‐D‐A character. It is worth noting that the absorption band of EDOT‐TPAO_4_ in film showed an obvious red shift compared to that in solution. This gradual redshift is attributed to the enhanced intra/inter‐ molecular charge transfer effect due to the aggregation in film and the similar results have been widely detected in recent work.^[^ ^42^, ^45^, ^48^, ^66^ ^]^

To investigate the presence of impurities in the samples, we conducted variable‐temperature UV–vis–NIR spectroscopy experiments. In the experiments, solution samples were placed in a 150 °C oil bath and subjected to thermal treatment for 10, 30, and 60 min, respectively. UV–vis–NIR spectra were immediately recorded after each thermal treatment step. Notably, the absorption spectra of samples treated for different durations exhibited high consistency across the measured wavelength range, with characteristic absorption peak positions remaining largely unchanged. (Figure 2b) This exceptional thermal stability indicated the absence of detectable heat‐sensitive impurities in the samples, strongly validating that our synthesis protocol yields high‐purity target products.

The UV–vis–NIR absorption spectra of EDOT‐TPAOMe_4_ and EDOT‐TPAO_4_ that EDOT‐TPAO_4_ possessed a narrower bandgap of 1.26 eV than its methoxy precursor of 2.74 eV. Its narrow bandgap nature provides a key advantage for photothermal applications as this narrow bandgap material EDOT‐TPAO_4_ powder can be excited by low‐energy near‐infrared light, such as a 1064 nm laser, for efficient photothermal conversion.^[^ ^69^ ^]^ The results further verify the effectiveness of the open‐shell ANAR radical modification strategy in regulating the band structure of photothermal materials. Moreover, the powder absorption spectrum of EDOT‐TPAO_4_ showed an extremely broad absorption band compared to its methoxy precursor (Figure 2g), indicating that it can more easily harvest more photons from sunlight. Compared with previously reported materials, EDOT‐TPAO_4_ demonstrated a significantly broader absorption bandwidth and exhibits exceptional photothermal performance under identical testing conditions.^[^ ^67^, ^68^ ^]^

### 2.3 Electrochemical Characterization via Cyclic Voltammetry

To investigate the electrochemical properties and frontier orbital energy levels of the materials, cyclic voltammetry (CV) measurements were conducted on both the methoxy precursor EDOT‐TPAOMe_4_ and the open‐shell radical derivative EDOT‐TPAO_4_ in dichloromethane and tetrahydrofuran solutions (Figure 2d,e). Additionally, the CV measurements were conducted on the film of EDOT‐TPAO_4_ in dichloromethane solution due to the poor solubility of its film in dichloromethane (Figure 2c). The highest occupied molecular orbital (HOMO) levels were estimated from the onset oxidation potentials, and the lowest unoccupied molecular orbital (LUMO) levels were calculated using the optical bandgaps obtained from UV–vis absorption spectra. In addition, we failed to conduct the CV test of the EDOT‐TPAOMe_4_ film due to its excellent solubility in the widely used solvents such as dichloromethane, acetonitrile, and THF.

EDOT‐TPAOMe_4_ exhibited a HOMO level of −4.69 eV, while EDOT‐TPAO_4_ displayed a slightly deeper HOMO at −4.75 eV. This downshift in HOMO is attributed to the electron‐withdrawing effect of the oxygen‐centered radical and the associated electron density redistribution in the open‐shell configuration, which reflects enhanced oxidative resistance and intrinsic electronic stabilization. This interpretation is well supported by the higher oxidation onset potential of EDOT‐TPAO_4_ (0.35 V for EDOT‐TPAO_4_ and 0.29 V for EDOT‐TPAOMe_4_), indicating electron‐withdrawing character of the open‐shell radical acceptor. Building on the aforementioned research, we conducted a 20‐cycle solution‐state voltammetry cycling test on the EDOT‐TPAO_4_ (Figure 2e). The results reveal exceptional stability surpassing expectations, with the electrochemical curves exhibiting negligible fluctuation post‐cycling. This phenomenon robustly confirms the structural robustness of radical materials.

### 2.4 Electron Spin Resonance Analysis

To investigate the open‐shell radical characteristics of EDOT‐TPAO_4_, electron spin resonance (ESR) spectroscopy was employed to characterize unpaired electron signals in powder samples. As shown in Figure 2f, under the equivalent molar amount powder sample testing conditions (0.02 mmol), the EDOT‐TPAO_4_ sample exhibited significantly higher ESR signal intensity with *g*‐factor value of 2.003, while its methoxy precursor EDOT‐TPAOMe_4_ showed none of ESR signal. This observation aligns well with the theoretical calculations revealing the complete absence of unpaired electrons in the molecular orbitals of EDOT‐TPAOMe_4_, confirming its closed‐shell chemical structure. Notably, EDOT‐TPAO_4_ demonstrates remarkable open‐shell radical character and stability in air, with the intense ESR signal in ambient conditions, and this radical formation can promote enhanced photothermal performance, as evidenced by our previous work.^[^ ^41^, ^42^, ^70^ ^]^

### 2.5 Electronic Excitation Analysis Based on TD‐DFT Methods

Time‐dependent density functional theory (TD‐DFT) calculations were conducted to elucidate the structural and electronic excitation characteristics of the demethylated precursor EDOT‐TPAOMe_4_ and the open‐shell radical EDOT‐TPAO_4_. All calculations were performed on isolated molecules without considering intermolecular interactions. Ground‐state geometry optimizations reveal that both EDOT‐TPAOMe_4_ and EDOT‐TPAO_4_ adopt twisted conformations (Figures S4 and S5, Supporting Information). S_1_‐state optimizations indicate slight structural relaxation of the molecular skeleton. The root‐mean‐square deviation (RMSD) between the optimized S_0_ and S_1_ geometries supports that the slight structural relaxations are related to the distal TPA moieties.

The singlet and triplet ground‐state energies and corresponding electronic excitation properties of EDOT‐TPAOMe_4_ and EDOT‐TPAO_4_ were quantitatively analyzed using TD‐DFT calculations (Table S3, Supporting Information). Results indicate that EDOT‐TPAOMe_4_ exhibits a typical singlet ground state with a large energy difference between the T_1_ and S_0_ states (*E*_T1_ ‐*E*_S0_ = 2.559 eV). In stark contrast, while EDOT‐TPAO_4_ also possesses a singlet ground state, its lowest triplet state (T_1_ state) is only 0.166 eV above S_0_. This near‐degeneracy, a characteristic commonly observed in open‐shell molecular systems, is expected to promote energetically favorable nonradiative decay pathways, thereby enhancing photothermal conversion efficiency.

**Figure 3:**
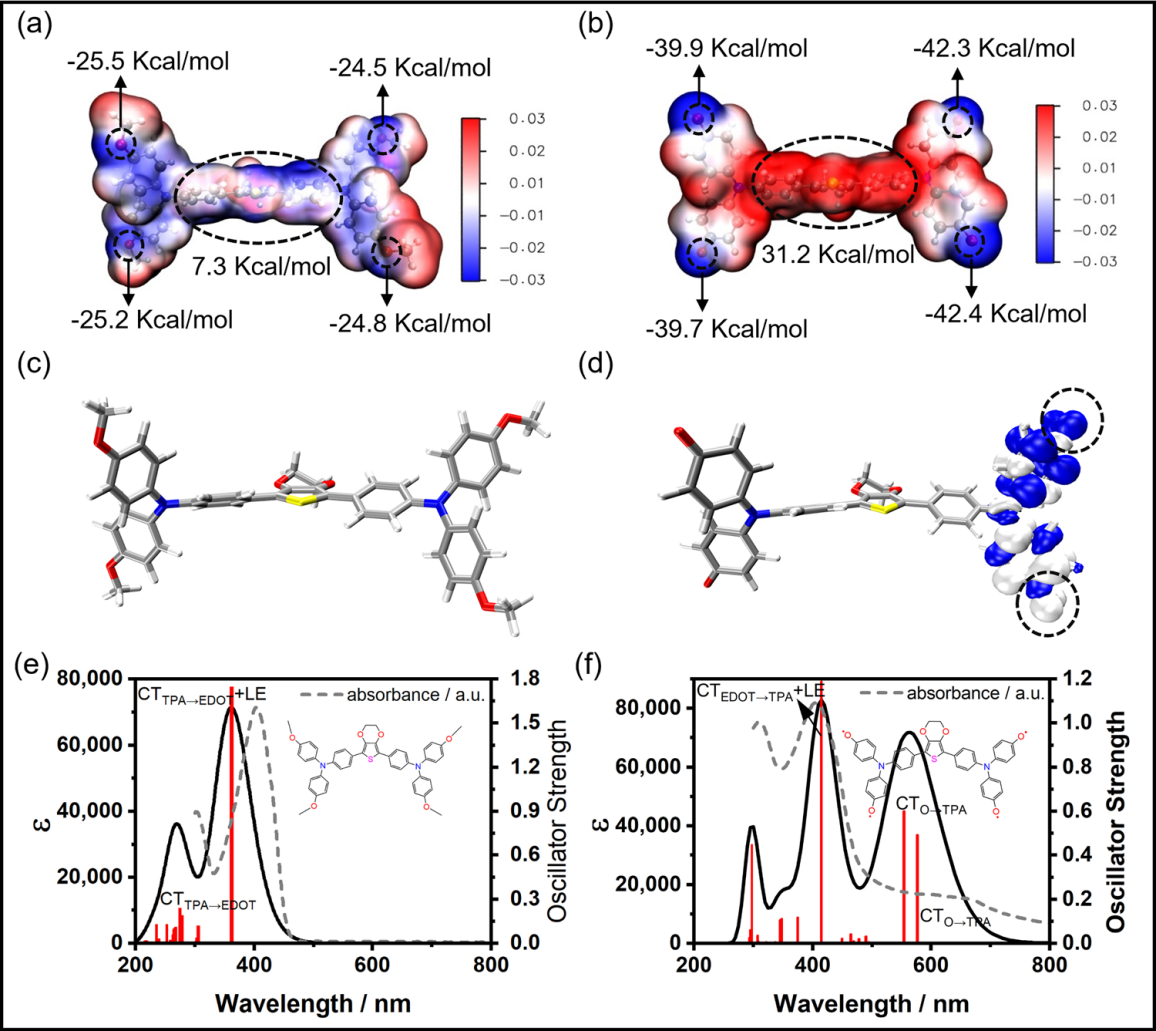
Electrostatic potential (ESP) maps of a) EDOT‐TPAOMe_4_ and b) EDOT‐TPAO_4_ in S_0_ geometry. The global minima and the average of fragment maxima were labeled by a black dashed circle, the unit is in kcal/mol. Isosurfaces of spin density for c) EDOT‐TPAOMe_4_ and d) EDOT‐TPAO_4_ in their S_0_ states. Blue and white isosurfaces correspond to positive and negative spin density, respectively. The isosurface levels are 0.0001 eÅ^−3^. Simulated electronic absorption spectra of e) EDOT‐TPAOMe_4_ and f) EDOT‐TPAO_4_ in their optimized S_0_ geometries. The spectral profiles are reconstructed by the GaussView software based on excitation energies of the major transitions and the corresponding oscillator strength (*f*). The grey dashed line represents the measured steady‐state UV–vis absorption with scaled intensity for comparison. All TD‐DFT calculations were conducted with Gaussian 16^[^ ^71^ ^]^ and performed on the TD‐(U)M06‐2X^[^ ^72^ ^]^/6‐31G(d,p)^[^ ^73^ ^]^ theoretical level.

Electrostatic potential (ESP) distribution on molecular surfaces was computationally investigated to elucidate ground‐state electronic features. ESP distributions were visualized on DFT‐optimized S₀ geometries using the VMD toolkit, with surface potentials mapped across discrete isovalues to distinguish regions of electron richness and deficiency (**Figure** **3**a,b). EDOT‐TPAOMe_4_ exhibits a relatively uniform ESP distribution across its molecular surface, with positive and negative potentials present within EDOT and TPAOMe_4_ moieties. Conversely, EDOT‐TPAO_4_ displays a significantly polarized ESP distribution in the ground state: the electron‐rich EDOT core exhibits positive electrostatic potential (red isosurface), while the oxygen‐centered radical termini show strongly negative potential character (blue isosurface). This analysis further confirms the A‐D‐A motif of EDOT‐TPAO_4_ formed by demethylation of the D'‐D‐D' type EDOT‐TPAOMe_4_. Notably, the global ESP minima of EDOT‐TPAO_4_ are centered at the oxygen radical sites. Spin density calculations corroborate this polarization, showing significant spin density localized at the distal oxygen radical sites in EDOT‐TPAO_4_ (Figure 3d), in contrast to its absence in EDOT‐TPAOMe_4_ (Figure 3c).

Vertical excitation analysis characterized the major electronic excitations of both compounds. Simulated electronic absorption spectra, derived from this analysis, align well with measured steady‐state spectra (Figure 3e,f). Owing to its D'‐D‐D' motif, EDOT‐TPAOMe_4_ exhibits a mixed LE_EDOT_ and CT_TPA→EDOT_ (also referred to as hybridized local and charge transfer, HLCT) character for its S_0_→S_1_ excitation. This transition has a large oscillator strength (*f* = 1.75) and a vertical excitation energy of 3.43 eV, corresponding to the major absorption band observed near 400 nm. In contrast, the lowest‐energy excitation (S_0_→S_1_) in EDOT‐TPAO_4_ shows CT character from EDOT unit to the oxygen radical sites of TPAO_4_ (CT_EDOT→TPAO4_), with an excitation energy of 2.15 eV. This corresponds to the measured low‐energy absorption band at ≈ 590 nm. The high‐energy excitation (S_0_→S_10_) exhibited mixed LE_EDOT_ and CT_EDOT→TPA_ (HLCT) character, with an energy of 2.99 eV, matching the measured major absorption band at 400 nm.

### 2.6 Photothermal Conversion Property

**Figure 4:**
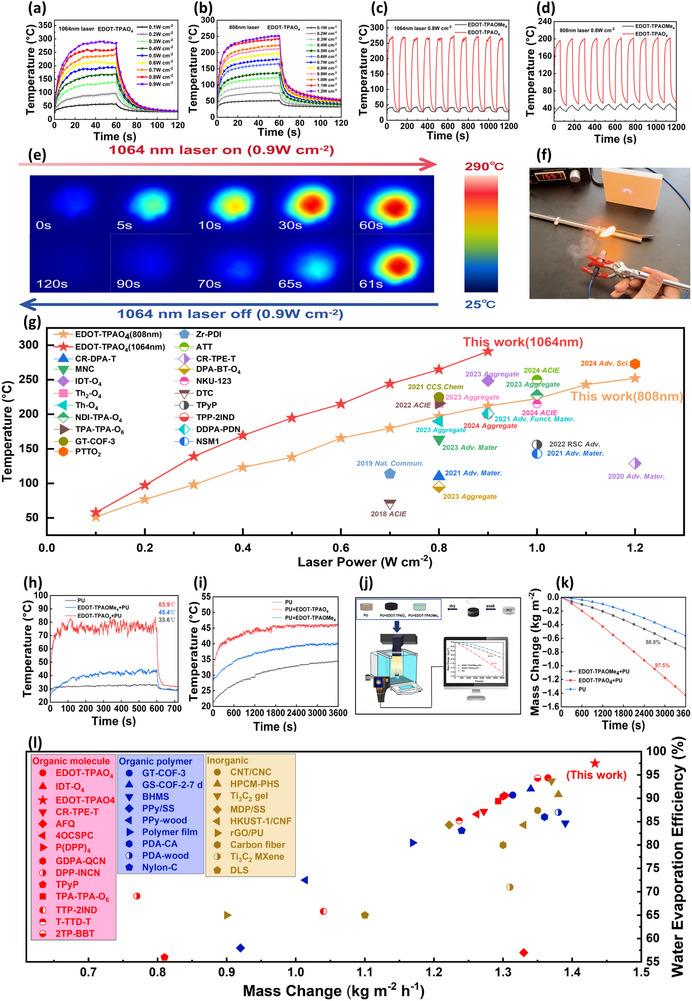
EDOT‐TPAO_4_ rising‐cooling curves at a laser wavelength of a) 1064 nm laser and b) 808 nm laser. The cyclic rising‐cooling curve of EDOT‐TPAO_4_ before and after demethylate on within 20 min at c) 1064 nm laser and d) 808 nm laser. e) Infrared thermal images of EDOT‐TPAO_4_ powder under 1064nm laser irradiation (0.9 W cm^−2^). f) Experiment with matchstick ignition coated with EDOT‐TPAO_4._ g) The comparison of photothermal conversion performance of reported materials (Tables S7 and S8, Supporting Information). h) The rising‐cooling curve of foams under 1 sunlight irradiation (1.0 kW m^−2^). i) The temperature changes of foams floating on water against sunlight irradiation time. j) Schematic diagram of water evaporation performance test under sunlight. k) Water evaporation curves of foams and radical‐loaded foams under simulated sunlight intensity of 1.0 kW m^−2^. l) The water evaporation rate and efficiency of the previous material compared with our work. (Table S9, Supporting Information).

Given its stable open‐shell radical behavior and broad powder absorption spectrum of EDOT‐TPAO_4_, we evaluated the photothermal conversion efficiency and infrared thermographic responses of EDOT‐TPAO_4_ and its precursor EDOT‐TPAOMe_4_ in powder form (15 mg per sample). Upon irradiation with near‐infrared lasers of 808 and 1064 nm, respectively, EDOT‐TPAO_4_ demonstrated markedly superior photothermal performance. At a power density of 1.2 W cm^−2^ (808 nm), the temperature of EDOT‐TPAO_4_ rapidly increased to 250 °C within 60 s, which represents as a high value in previously reported pure organic photothermal materials, while EDOT‐TPAOMe_4_ only reached 52 °C under the same conditions (**Figure** **4**b; Figure S12, Supporting Information).

Notably, as the powder absorption of EDOT‐TPAO_4_ increased with wavelength in the near‐infrared region, further photothermal evaluation was conducted under 1064 nm irradiation. At a lower power density of 0.9 W cm^−2^, EDOT‐TPAO_4_ exhibited a rapid temperature rise to 290 °C within 60 s (Figure 4a), consistent with its broad and red‐shifted absorption profile in the powder state (Figure 4e). Compared to 808 nm laser, 1064 nm irradiation offers enhanced beam quality, deeper tissue penetration, improved thermal controllability, and greater suitability across diverse application scenarios, including high‐precision industrial processing, deep‐tissue medical treatment, and military technology. These findings underscore the promising potential of EDOT‐TPAO_4_ for advanced photothermal applications.^[^ ^38^, ^51^, ^74^ ^]^

To further assess its thermal durability, ten consecutive heating–cooling cycles were performed at 0.8 W cm^−2^ (Figures 4c,d and 1c). EDOT‐TPAO_4_ maintained excellent photothermal activity and thermal stability, with rapid temperature escalation during each irradiation cycle and no significant loss in performance after repeated use. The thin layer chromatography results (Figure 1c) and the ^1^H‐NMR spectrum (Figure S17, Supporting Information) of the EDOT‐TPAO_4_ sample before and after 10 photothermal cycles further confirms the high stability of this radical and indicate minimal thermal fatigue and negligible photobleaching effects. Figure 4g compares the photothermal performance of EDOT‐TPAO_4_ with that of previously reported organic materials, revealing its superior efficiency; detailed photothermal data are provided in Table S7 (Supporting Information). Moreover, a linear correlation was observed between maximum temperature and laser power (Figures S14 and S15, Supporting Information), further confirming the efficient and predictable photothermal response of EDOT‐TPAO_4_.

### 2.7 Photothermal‐Driven Laser Ignition Enabled by EDOT‐TPAO_4_

Leveraging the superior photothermal conversion properties of the EDOT‐TPAO_4_ compound, this study explored its application in non‐contact laser ignition. Traditional wood ignition methods, such as direct flame or friction, suffer from poor spatial controllability and are inadequate for precision ignition scenarios. A 3.0 mg/mL ethanol solution of EDOT‐TPAO_4_ was prepared, and commercial wooden matchsticks were immersed and dried. Ignition tests were performed using an 808 nm laser at 1.3 W cm^−2^ with a vertical irradiation distance of ≈5 cm (Figure 4f; Movie S1, Supporting Information). Upon targeting the match head with the laser beam, ignition occurred within 10 s. In contrast, untreated matchsticks failed to ignite under identical conditions. These results strongly support the feasibility of EDOT‐TPAO_4_ for photothermal‐driven precision ignition.^[^ ^75^ ^]^

### 2.8 Solar‐Driven Water Evaporation Performance

Given that the absorption spectrum of EDOT‐TPAO_4_ spans the entire solar range, we investigated its solar‐driven interfacial water evaporation capability. In the experiment, 25 mg of EDOT‐TPAO_4_ and EDOT‐TPAOMe_4_ were respectively dissolved in methylene chloride and ethanol, then uniformly coated onto polyurethane (PU) sponges and dried to obtain powder‐loaded sponges. The fabrication process is illustrated in Figure 4j. Under simulated solar irradiation (1 kW m^−2^, 10 min), the surface temperatures of EDOT‐TPAOMe_4_ and EDOT‐TPAO_4_ loaded PU reached 45.3 and 83.9 °C, respectively (Figure 4h).

After 1 h of sunlight exposure (Figure 4i), wet PU, EDOT‐TPAOMe_4_+PU, and EDOT‐TPAO_4_+PU exhibits surface temperatures of 34.7, 40.4, and 46.4 °C, respectively. The water mass loss was recorded every 5 min to assess evaporation efficiency. As shown in Figure 4k, EDOT‐TPAO_4_+PU exhibited a significantly steeper mass loss slope than EDOT‐TPAOMe_4_+PU. The corresponding water evaporation rate and solar conversion efficiency were calculated to be 1.433 kg m^−2^ h^−1^ and 97.5% for EDOT‐TPAO_4_+PU, and 0.781 kg m^−2^ h^−1^ and 53.1% for EDOT‐TPAOMe_4_+PU (calculation details in Table S6, Supporting Information). Compared with inorganic and polymeric materials, EDOT‐TPAO_4_ offers notable advantages, including a simple synthetic route, excellent reproducibility, and superior water evaporation efficiency. Furthermore, its good solubility in ethanol and DMSO aligns with requirements for biomedical applications.^[^ ^27^, ^41^, ^76^ ^]^ Figure 4l further compares our results with previous reports on small‐molecule, polymeric, and inorganic evaporators.

### 2.9 Excited‐State Dynamics Resolved by Femtosecond Spectroscopy

**Figure 5:**
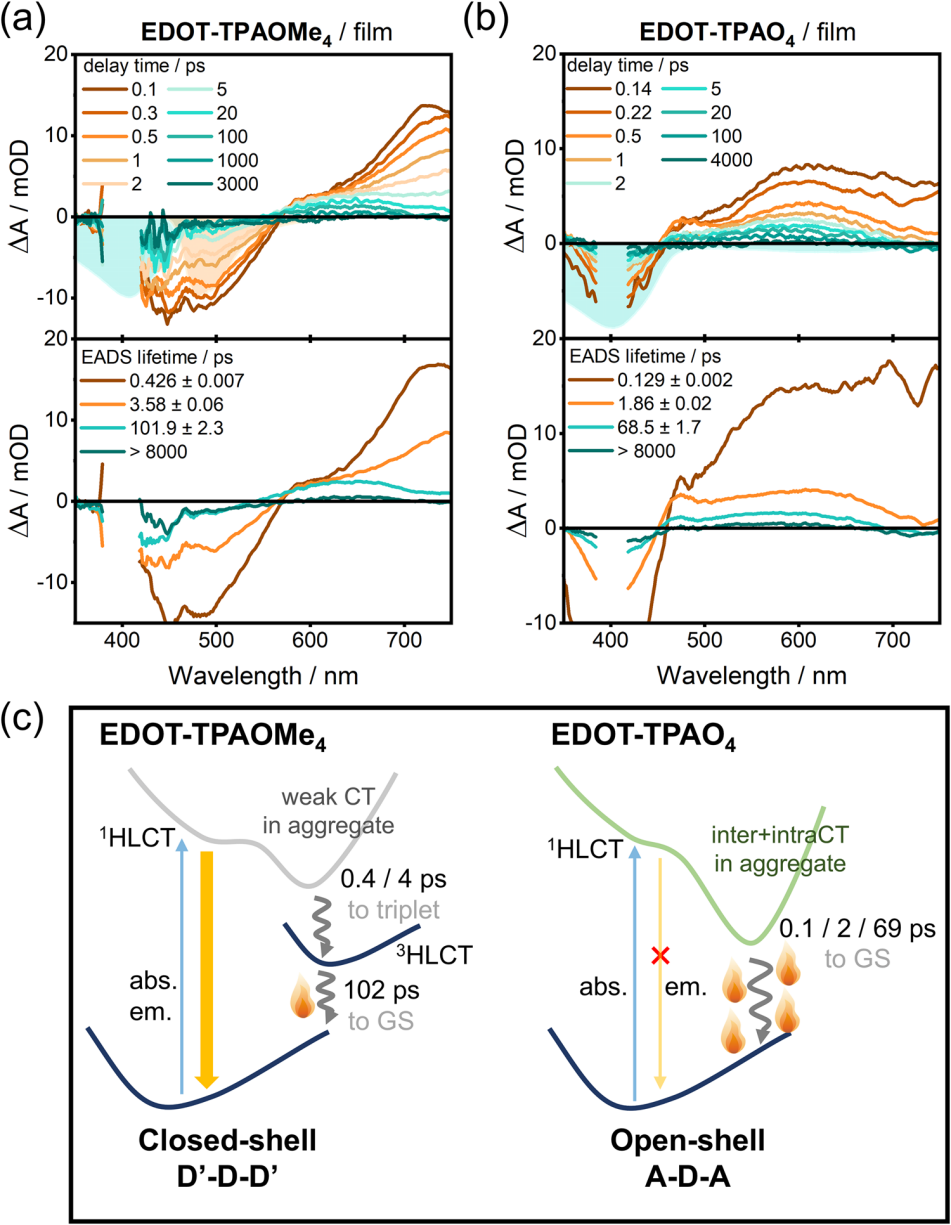
Time evolution of femtosecond TA spectra of a) EDOT‐TPAOMe_4_ and b) EDOT‐TPAO_4_ in neat films upon excitation at 400 nm in the 8‐ns time window. The cyan and orange shaded areas represent the corresponding stationary absorption and fluorescence spectra. Evolution‐associated difference spectra (EADS) obtained from the global analysis based on a sequential model are shown in the lower panels below the corresponding TA spectra. The fitting time constants are shown in the legends. c) The relaxation dynamics model of EDOT‐TPAOMe_4_ and EDOT‐TPAO_4_.

Efficient light‐to‐heat conversion depends critically on excited‐state quenching. To elucidate these pathways, the excited‐state dynamics of neat films of EDOT‐TPAOMe_4_ and EDOT‐TPAO_4_ were investigated by femtosecond time‐resolved transient absorption (fs‐TA) spectroscopy (**Figure** **5**). For EDOT‐TPAOMe_4_ upon 400 nm excitation (Figure 5a), the 0.1‐ps fs‐TA spectrum, immediately following instrument response, reveals three features: ground‐state bleach (GSB) below 450 nm, stimulated emission (SE) centered at 480 nm, and broad excited‐state absorption (ESA) with peaks near 590 and 720 nm. This signature corresponds to the ^1^HLCT state identified by TD‐DFT calculations. On the nanosecond time scale, ^1^HLCT absorption features gradually quench while a long‐lived species emerges within the 8‐ns time window, assigned to the ^3^HLCT state (confirmed by nanosecond transient absorption (ns‐TA) measurement in Figure S11 (Supporting Information). EDOT‐TPAO_4_ undergoes analogous Franck‐Condon excitation at 400 nm. Its initial 0.14‐ps fs‐TA spectrum (Figure 5b) exhibits a similar ^1^HLCT profile to EDOT‐TPAOMe_4_, while the SE band is not observed due to aggregate emission quenching. Crucially, EDOT‐TPAO_4_ shows continuous spectral evolution in picosecond time scale without long‐lived species formation. As its ^1^HLCT state is a high‐lying excited state, internal conversion to the low‐lying ^1^CT_O→TPA_ state dominates the early excited‐state relaxation. The rapid ESA decay in ca. 100 ps signifies efficient non‐radiative decay, enabling efficient energy dissipation through thermal pathways that boost photothermal conversion efficiency.

Although excited‐state relaxation via structural relaxation is undoubtedly a continuous process (especially for EDOT‐TPAO_4_), we employ a state‐to‐state evolution model to mimic the excited‐state quenching dynamics. Assuming a sequential evolution involving four discrete excited species, we performed global spectral analysis to obtain evolution‐associated difference spectra (EADS) and corresponding rate constants (Figure 5a,b, lower panels). For EDOT‐TPAOMe_4_, the first and second EADS components, exhibiting a similar spectral profile but distinct amplitude, are assigned to the ^1^HLCT state. The third and fourth EADSs were assigned to the ^3^HLCT state according to the ns‐TA measurements with oxygen‐sensitive excited‐state dynamics (Figure S11, Supporting Information). In contrast, for EDOT‐TPAO_4_, the first EADS component is assigned to the ^1^HLCT state with a shorter lifetime (0.129 ps) than EDOT‐TPAOMe_4_. This rapidly converts to a short‐lived ^1^CT_O→TPA_, characterized by a biexponential decay (lifetime: 1.86 and 68.5 ps).

Consequently, we established the excited‐state relaxation model of EDOT‐TPAO_4_ and EDOT‐TPAOMe_4_ shown in Figure 5c. For closed‐shell EDOT‐TPAOMe_4_ (D'‐D‐D' motif), in the aggregate state, it undergoes rapid intersystem crossing (≈4 ps) to generate ^3^HLCT. This triplet state efficiently quenches within hundreds of picoseconds, enabling efficient photothermal conversion. For open‐shell EDOT‐TPAO_4_ (A‐D‐A motif), following excitation to ^1^HLCT, it undergoes an internal conversion to the ^1^CT_O→TPA_ arising from the demethylation‐induced structural modification. The ^1^CT_O→TPA_ state (involving both intramolecular and intermolecular CT interaction in the aggregate state) undergoes direct excited‐state quenching to the hot ground state. These fundamental differences in the excited‐state quenching mechanims lead to significantly enhanced photothermal performance in EDOT‐TPAO_4_.

## 3 Conclusion

In summary, we developed a high‐performance organic photothermal material design strategy through targeted integration of open‐shell oxygen‐centered radical acceptors into an acceptor–donor–acceptor (A‐D‐A) framework. An open‐shell aromatic nitric acid radical EDOT‐TPAO_4_ was readily prepared in one step demethylation of commercially available EDOT‐TPAOMe_4_ with high yield of 76%. Comparing with the wide bandgap EDOT‐TPAOMe_4_, the A‐D‐A structured EDOT‐TPAO_4_ exhibited a much lower bandgap of 1.26 eV and an ultrabroad absorption spectrum in powder from 300 to 2500 nm. Interestingly, EDOT‐TPAO_4_ processed high electrochemical stability in air and unexpected electron conductivity of 0.02 S cm^−1^ due to its open‐shell character. Compared with previous reported pure organic photothermal conversion materials, EDOT‐TPAO_4_ exhibits remarkable photothermal conversion efficiency. Notably, EDOT‐TPAO_4_ achieved 290 °C within 60 s under 1064 nm laser irradiation with power density of 0.9 W cm^−2^, which is significantly higher than 45 °C of its precursor EDOT‐TPAOMe_4_. EDOT‐TPAO_4_ show higher photothermal conversion efficiency under 1064 nm laser irradiation than the traditional 808 nm, which is consistent with the absorption spectra in powder. Moreover, this material exhibits an exceptional solar‐driven water evaporation rate of 1.433 kg m^−2 ^h^−1^ and high energy conversion efficiency, which surpass those of most reported organic materials, highlighting the synergistic benefits of the open‐shell radical. TD‐DFT calculation and femtosecond spectroscopy the distinct electronic structure and excited‐state dynamics between EDOT‐TPAO_4_ and EDOT‐TPAOMe_4_. Ultrafast excited‐state nonradiative quenching of EDOT‐TPAO_4_ in picosecond timescale underly this excellent photothermal conversion. This work provides a new idea to design photothermal materials and organic semiconductors in the future. The synthesis of open‐shell radical materials with higher photothermal conversion efficiency and electron conductivity, and the exploration of their applications in optoelectronic devices andare in urgent progresss in our laboratory.

## Conflict of Interest

The authors declare no conflict of interest.

## Author Contributions

H.Z. and Y.Y. contributed equally to this work; Y Li conceived the idea and supervised the overall project;Y.L. and H.Z. synthesized the new compounds in the manuscript and drafted the manuscript; Z.K. and Y.Y. contributed to all theoretical calculations and supplemented the manuscript; J.H., S.S., and Z.L. contributed to the experimental investigation; Q.D., L.C., and Y.C. contributed to the application studies of the compounds; Q.D., Z.K., and Y.L. contributed to the revision of the article; All authors contributed to the general discussion.

## Supporting information



Supporting Information

Supplemental Movie 1

## Data Availability

The data that support the findings of this study are available in the supplementary material of this article.
