# Supplementary material for: Stable Ultrabroad‐Absorbing Radical Achieves Efficient NIR‐II Photothermal Conversion via Facile Synthesis
Source: Adv Sci (Weinh). 2025 Sep 3;12(43):e13587. doi: 10.1002/advs.202513587 (PMC12631838; doi:10.1002/advs.202513587)
Supplement: Supplementary file 1 — Supporting Information [file ADVS-12-e13587-s001.docx]

**Supporting Information**

**Stable Ultrabroad-Absorbing Radical Achieves Efficient NIR-II Photothermal Conversion via Facile Synthesis**

Haozhe Zhang^† [a]^, Yuhang Yang^† [b]^, Jiaxing Huang^[a]^, Luotai Chen^[c]^, Yongyan Cui^[c][d]^, Zhiyuan Lu^[a]^, Shishi Shen^[a]^, Zixi Liu^[b]^, Hongwei Song^[a]^, Zhuoran Kuang^*[b]^, Qianxi Dang^*[c][d]^ and Yuan Li^*[a]^

†: These authors contributed equally.

^[a]^ State Key Laboratory of Luminescent Materials and Devices, Institute of Polymer Optoelectronic Materials and Devices, School of Materials Science and Engineering, South China University of Technology, Guangzhou 510640, P. R. China, E-mail: [celiy@scut.edu.cn](mailto:celiy@scut.edu.cn)

^[b]^ State Key Laboratory of Information Photonic and Optical Communications, and School of Physical Science and Technology, Beijing University of Posts and Telecommunications (BUPT), Beijing 100876, P. R. China. E-mail: [kuang@bupt.edu.cn](mailto:kuang@bupt.edu.cn)

^[c]^ Department of Plastic and Reconstructive Surgery, Peking University Shenzhen Hospital, Shenzhen 518036, Guangdong, P. R. China. E-mail: dangqianxi@pkuszh.com

^[d]^ Guangdong Provisional Key Laboratory of Functional Oxide Materials and Devices, Southern University of Science and Technology, Shenzhen 518055, Guangdong, P. R. China.

**Contents**

1.Reagents and materials .1

**Table S1**1

2.Methods .1

**2.1 Table S2**1

**2.2 Detailed synthetic route for the radicals**2

**2.3 Molecular synthesis and characterization**2

**2.4 UV-vis absorption and fluorescence measurements**3

**2.5 Cyclic voltammograms of radicals**3

**2.6 Photothermal preperties**3

**2.7 Preparation of material-loaded sponge**4

**2.8 Water evaporation experiment**4

**2.9 Water evaporation conversion efficiency**4

**2.10 Femtosecond Transient Absorption Spectral Measurements.**4

**2.11 Nanosecond Transient Absorption Spectral Measurements.**5

**2.12 Quantum Calculation**5

3.The ^1^H-NMR spectra of compounds.6

**Figure S2**.6

4.The MS spectra of compounds6

**Figure S3**.6

5. Supplemental TD-DFT Calculations _._7

**Figure S4**.7

**Figure S5**.8

**Figure S6**.9

**Figure S7**.10

**Figure S8**.10

**Table S3**.11

**Table S4**.11

**Figure S9**.12

6. Supplemental transient absorption spectra13

**Figure S10**13

**Figure S11**.14

7.Supplementary photothermal properties.15

**Figure S12**.15

**Figure S13**.15

**Figure S14**.16

**Figure S15**16

**Figure S16**17

**Figure S17**17

**Figure S18**17

**Figure S19**18

8.Characterization comparison of EDOT-TPAOMe_4_ and EDOT-TPAO_4._19

**Table S5**.19

9.The powder absorption of organic small molecules.20

**Figure S20**20

10.The calculation of the efficiency for solar to vapor generation.21

**Table S6**21

11.Comparison of photothermal properties of different materials.22

**Table S7**22

**Table S8**23

12.Comparison of water evaporation properties of different materials26

**Table S9**26

13.Reference27

##

## Reagents and materials

Table S1. Commercially available reagents and materials in our research

| **Materials** | **CAS** | **Purity(%)** | **Producer** |
| --- | --- | --- | --- |
| EDOT-TPAOMe_4_ | 1622008-73-4 | 98% | SuZhou SunaTech Inc |
| Boron tribromide | 10294-33-4 | 99.9% | Shanghai Macklin Biochemical Technology Co., Ltd |
| DMSO | 67-68-5 | 99.8% | Energy Chemical, China |
| Dichloromethane | 75-09-2 | 99.9% |  |
| Ethyl acetate | 141-78-6 | 99.9% |  |
| Ethanol | 64-17-5 | 99.8% |  |

1. **Methods**

**2.1 Table S2. The measurement and equipment in our research**

| **Test method** | **Equipment specification and remarks** |
| --- | --- |
| ^1^H /^13^C nuclear  magnetic resonance | Bruker Avance 400 or 500 MHz spectrometer |
| UV-Vis-NIR absorption spectra | UV-3600 (Shimadzu Co., Japan) |
| Mass Spectrum | MALDI-TOF-MS |
| Cyclic voltammetry | CHI660E, China  [Hg/Hg2Cl2 (3M KCl solution) electrode;  Carbon-glass electrode; Pt line electrode;  Electrochemistry Workstation] |
| Powder Absorption | Lambda 950 spectrophotometer |
| Water Evaporation Test | Solar simulator with an optical filter for the standard  AM 1.5 G spectrum (CEL-S500/350/150); IR  thermal camera (FLIR E4) |
| Electron spin  resonance spectroscopy | Bruker ELEXSYS E500 spectrometer |
| Powder Absorption | Powder Absorption |
| Thermogravimetric analysis | NETZSCH TG 209 |
| Photothermal Property | 808 nm Fiber Coupled Laser (Model: MW-GX-808, Changchun Laser Optoelectronics Technology Co., Ltd.);  1064 nm Fiber Coupled Laser(Model: PSU-H-LED,  Changchun New Industries Optoelectronics Tech. Co., Ltd;  IR thermal camera (FLIR E4) |
| Femtosecond Transient Absorption Spectral Measurements. | Transient absorption spectrometer (Harpia-TA), sapphire laser (Astrella, Coherent), optical parametric amplifier (TOPAS-C, Light Conversion) |
| Nanosecond Transient Absorption Spectral Measurements. | Spectrometer (Time-Tech Spectra),supercontinuum laser (LEUKOS-DISCO, French) |

**2.2 Detailed synthetic route for the radicals**


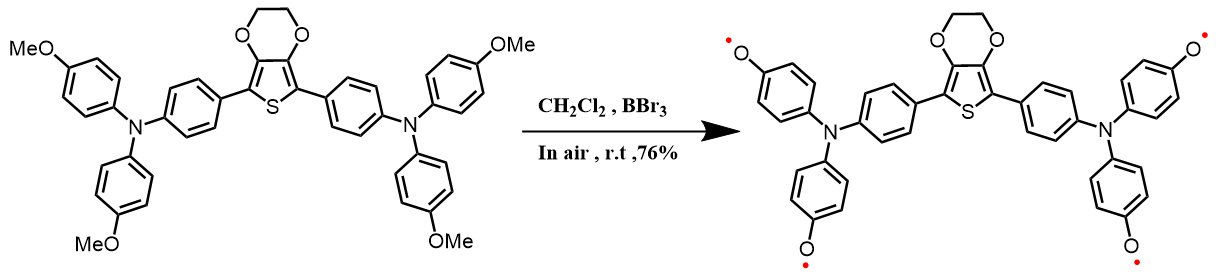


**Figure S1.** Synthesis of EDOT-TPAO_4_

Synthesis of **EDOT-TPAO_4_**

EDOT-TPAOMe_4_ (Synthetic routes to EDOT-TPAOMe_4_ can be found in previous reported work. ^[1]^) (500 mg) is dissolved in dried methylene chloride (20 mL) and placed into magnets and stirred on a stirring table. Drop 99.9 % pure boron tribromide (0.2 mL) into the solution at room temperature and plug the mouth of the reaction bottle with a glass stopper. The mixture was stirred at room temperature for six hours, and solid products precipitated. Deionized water is then added drop by drop to quench the boron tribromide, while the solid product continues to precipitate. Subsequently, the solid product was separated by filtration and repeatedly washed with deionized water to obtain the crude demethylated product EDOT-TPAO_4._ Then, the crude product was purified by silica gel column chromatography (ethanol:ethyl acetate = 1:1), which finally affords 380 mg of dark green powder with a yield of 76 %. **^1^H NMR (400 MHz, DMSO-d6) δ 9.35 (s, 1H), δ 7.43(s,1H) δ 6.91-6.72 (d, 5H)**

**δ 4.31 (s, 1H).**

**2.3 Molecular synthesis and characterization**

Molecular synthesis can be obtained in **Fig. S1**. All the chemical structures of methoxy precursors and radicals were carefully characterized by ^1^H/^13^C nuclear magnetic resonance (NMR) and mass spectrometry (**Fig. S2-S3**). 7 mg dry sample for ^1^H-NMR was placed in a nuclear magnetic tube and then perform the test (DMSO-d6 for radical species). The mixture of materials with 4,6-Dinitro-2-sec-butylphenol (matrix) and sodium trifluoroacetate was dropped on the substrate and dried in mass spectrometry test, and the mass spectrometry was measured in positive mode. The UV-vis NIR spectra of film and solution were recorded on a UV-3600 (Shimadzu Co., Japan). The UV-vis NIR spectra of powder samples were conducted on S3Lambda 950 spectrophotometer. Cyclic voltammograms (CV) results were obtained in CHI660E. Electron spin resonance (ESR) spectroscopy of powder samples (0.02 mmol) were measured on Bruker ELEXSYS E500 spectrometer. The variable temperature electron spin resonance (VT-ESR) of powder samples (0.02 mmol) were measured on Bruker A300-10/12 (Germany). The solar-driven water/seawater evaporation experiments were conducted on Solar simulator with an optical filter for the standard AM 1.5 G spectrum (CEL-S500/350/150). In this experiment, the irradiation intensity is one solar irradiation intensity (1.0 kW m^-2^). The surface temperature of the foam was recorded by an IR thermal camera (FLIR E4). The weight loss of water was measured by an electronic mass analytical balance (Sartorius BSA224S).

**2.4 UV-vis absorption and fluorescence measurements**

The absorption spectra of powder samples were obtained via Lambda 950 spectrophotometer coupled with a sensitive integrating sphere. The instrument is zeroed by a blank barium sulfate, and then a layer of powder sample is evenly pressed on the barium sulfate to obtain the absorption spectrum of the powder state. The absorption spectra of materials in solution or film state were obtained from UV-3600 (Shimadzu Co., Japan). The solution samples were tested with a concentration of 10-5 mol L^-1^ in DMSO and the spin-coated films were obtained by spinning the material onto silica glass with a rotational speed of 2000 rpm, 30 s.

## 2.5 Cyclic voltammograms of radicals

All the CV curves were measured in air in the dry dichloromethane solution containing 0.1 M nBu4NPF6 as a supporting electrolyte and the scan rate was 0.1 Vs^-1^. The carbon-glass electrode, platinum electrode, and saturated calomel electrode were applied as working electrode, counter electrode, and reference electrode, respectively. The target radical compounds were coated on the carbon-glass electrode to form the film. Potential values are reported with the saturated calomel electrode as the reference electrode using the Fc^+^ /Fc couple (0.37 V) as an internal standard.

**2.6 Photothermal preperties**

After being fully dired at 80 °C, 15 mg of each radical was placed evenly and compactly on the surface of the weighting paper. The photothermal conversion properties of radical species were characterized under an 808 nm and 1064 nm laser with a power density of 0.8 W cm^-2^ (strictly control the size of the light spot to 1 cm^2^

to obtain accurate light intensity). The temperature-time curve can be obtained by irradiating the material for 60 seconds and then turning off the laser. Similarly, the anti-photobleaching properties of precursors and radicals are characterized through above operations.

**2.7 Preparation of material-loaded sponge**

A commercially available polyurethane sponge (1.2 cm radius) was utilized as a carrier. The radical-loaded PU foam was obtained by setting the sponge in ethanol solution with 30 mg radical. Then, the wet radical-loaded PU was placed in an oven for 3 hours under 60 °C.

**2.8 Water evaporation experiment**

In the environment of 24-25 °C and 45-55 % relative humidity, the water-filled beaker with sponge is placed on the analytical balance. Under the irradiation of simulated sunlight with an intensity of 1 kW m^−2^(one sun), we recorded the balance reading every five minutes (representing the lost water mass). After one hour, the twelve data points obtained can be used to fit the water evaporation curves.

**2.9 Water evaporation conversion efficiency (η)**

The η in water evaporation was calculated as the following formula^[7]^:

*η* = *ṁ h_LV_* /*Copt P_0_*

**Equation S1.** Calculation formula for water evaporation conversion efficiency. The specific calculation details can be referred to **Table S4**

**2.10 Femtosecond Transient Absorption Spectral Measurements.**

Femtosecond time-resolved transient absorption spectra were measured using a commercial transient absorption spectrometer (Harpia-TA, Light Conversion). Briefly, fundamental pulses are derived from an amplified femtosecond Ti: sapphire laser (Astrella, Coherent). The laser delivers 40 fs pulses at 1kHz and the output is split for white-light continuum generation and optical pumping. The white-light continuum is used as a broadband optical probe from the near-UV to the visible region. It is generated by focusing the fundamental laser beam into a 2 mm thick CaF_2_ plate, which is oriented and continuously shifted perpendicularly. The required pumping pulse is obtained by an optical parametric amplifier (TOPAS-C, Light Conversion). The pump and probe beams were overlapped on a 1 mm thick sample cell and the included polarization angle was set to the magic angle (54.7°) to record the isotropic response. Transient absorption is calculated from consecutive pump-on and pump-poff measurements and averaged over 1000 shots. UV-Vis absorption spectra of the samples are measured before and after every measurement in a spectrophotometer. No significant photo-degradation was observed after the TA measurements. The femtosecond time-resolved differential absorbance data were analyzed by using R-package TIMP software with the graphical interface Glotaran^[2]^ and CarpetView (Light Conversion). In the global target analysis, the differential absorbances Δ*A* (*t*, *λ*) are decomposed as a superposition of several principal spectral components *ε_i_* (*λ*) weighed by their concentrations *c_i_* (*t*) ^[3]^.

$$\begin{aligned} \Delta A\left( t,\lambda\right)= \sum_{i=1}^{n} c_{i}\left( t \right)\varepsilon_{i}\left( \lambda\right)\#\left( 1 \right) \end{aligned}$$

**2.11 Nanosecond Transient Absorption Spectral Measurements.**

The ns-TA spectra were measured by a commercial spectrometer (Time-Tech Spectra). The generation of the pump beam is the same as that in fs-TA. The probe beam was generated from a supercontinuum laser (LEUKOS-DISCO, French) with the spectral region from 350 to 1800 nm, the repetition rate is 2 kHz, and pulse width is 700 ps to 1 ns. There is no photodegrading after ns-TA experiments by checking the steady-state absorption spectra.

**2.12 Quantum Calculation.**

All simulations were implemented for isolated molecules using the Gaussian 16 software package^[4]^. The calculations are performed using density generalized function theory (DFT) and time-dependent DFT (TD-DFT) methods. The geometric optimization and excitation energy calculation of the ground state (S_0_), the first excited singlet state (S_1_) and the triplet state (T_0_) were using DFT/UDFT at the high-nonlocality hybrid functional M06-2X ^[5]^ with the 6-31G (d,p)^[6]^ basis set. In all the cases, frequency analysis was made after geometry optimization to ensure the convergence to an energy minimum. TD-DFT calculations of the UV-vis absorption spectra were done at S_0_ optimized geometries using M06-2X /6-31G (d,p). Spin density, electrostatic Potential (ESP)^[7]^ and root mean square deviation (RMSD) were conducted by the Multiwfn and VMD program^[8-9]^.

1. **The ^1^H-NMR spectra of compounds**

**
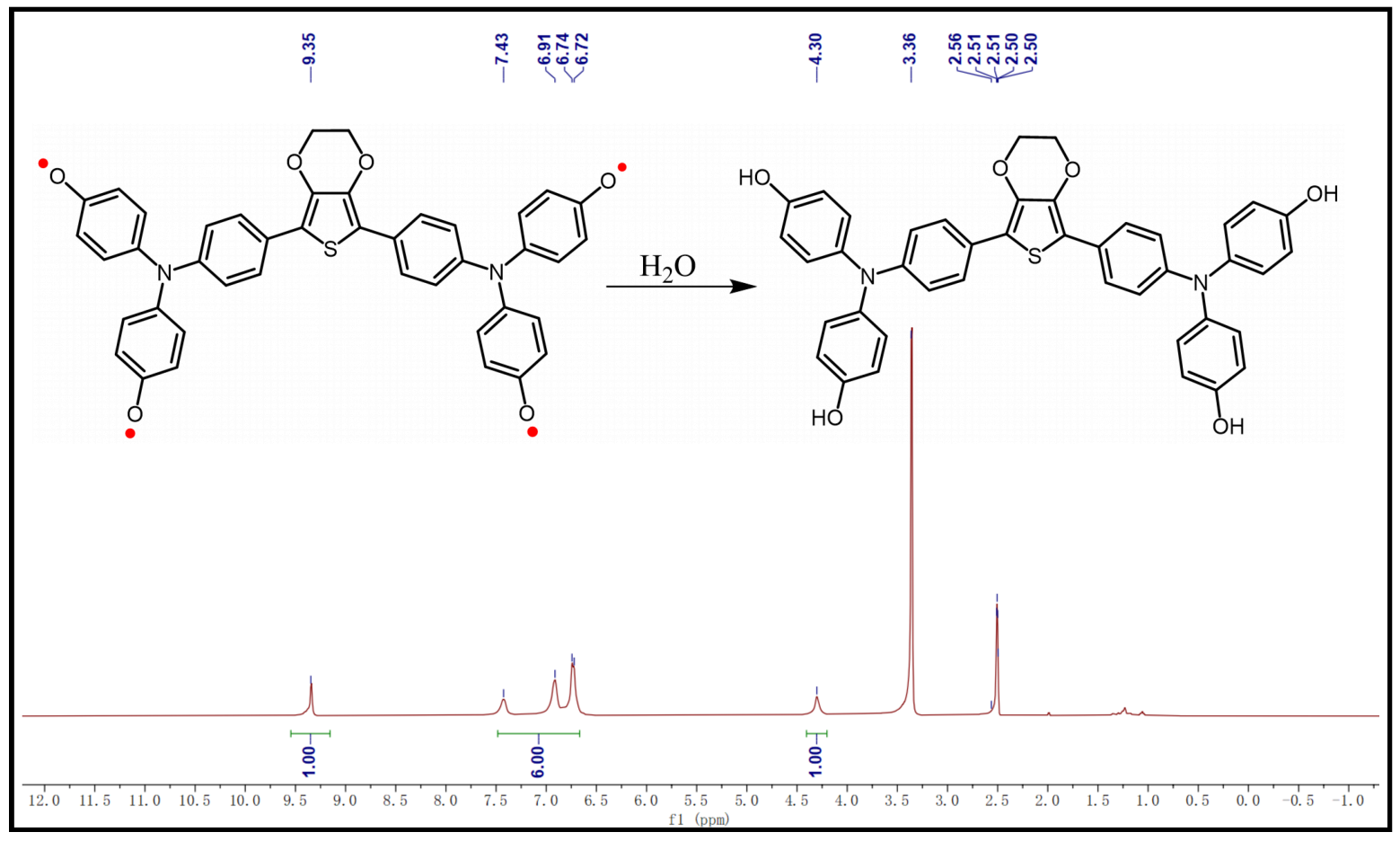
**

**Figure S2.** ^1^H-NMR spectrum of EDOT-TPAO_4_

1. **The MS spectra of compounds**

**
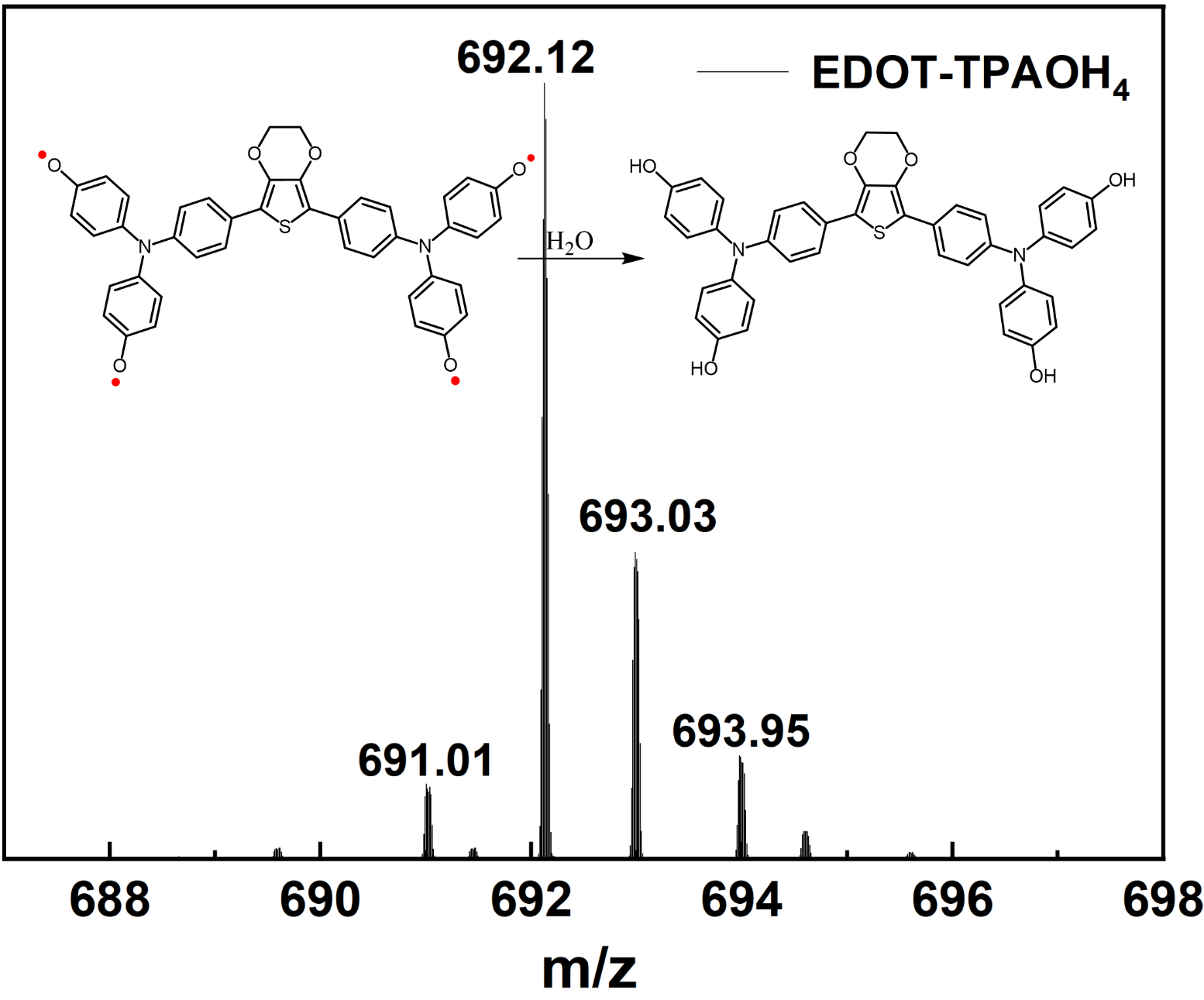
**

**Figure S3.** High-resolution MALDI-TOF-MS of EDOT-TPAO_4_ in positive mode. Calcd for C_42_H_32_N_2_O_6_S: m/z: 692.20. Found: 692.12 (For radical structures, they combine with trace amounts of water during mass spectrometry testing to form hydroxyl radicals). ^[10-12]^

1. **Supplemental TD-DFT Calculations**


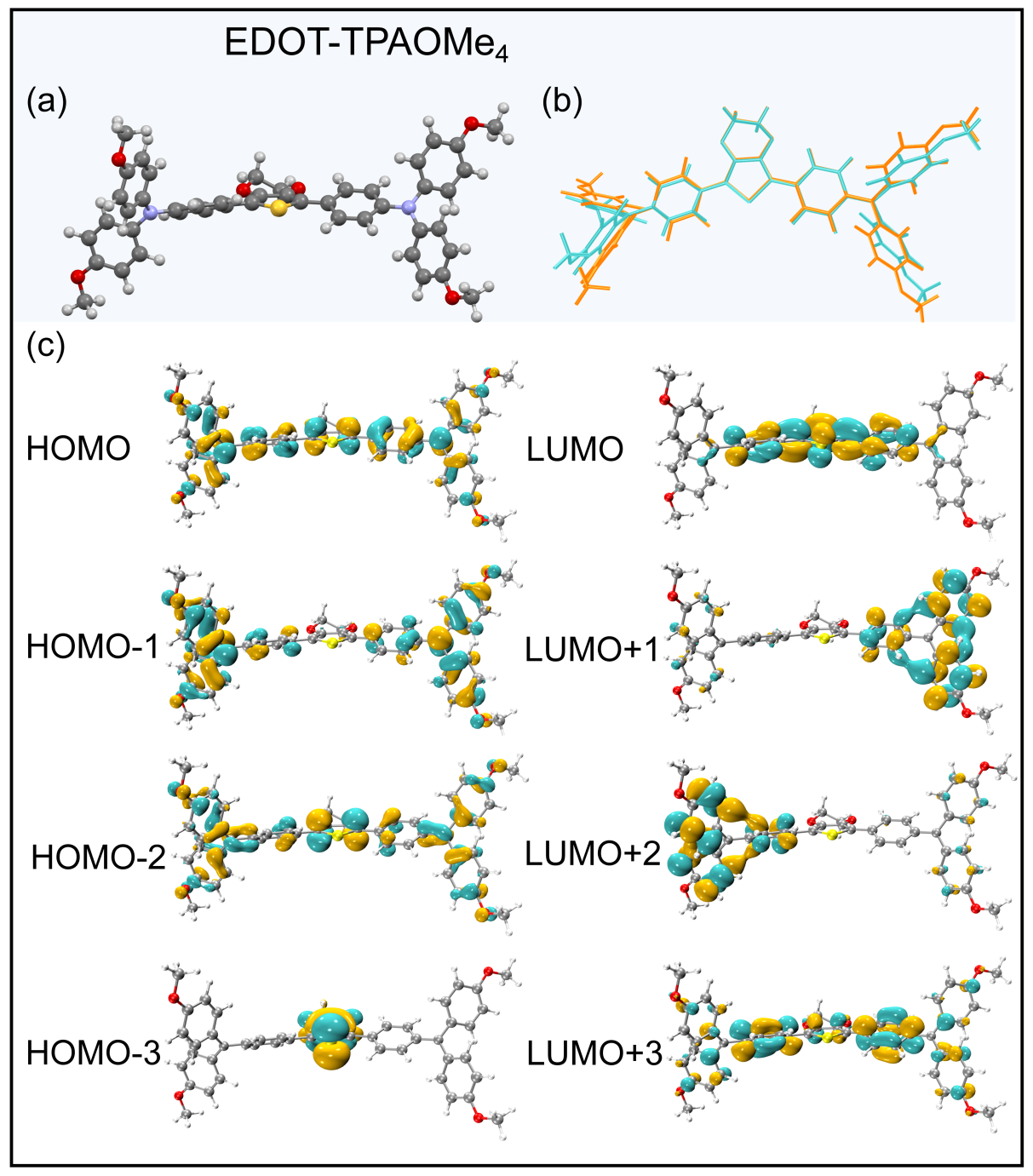


**Figure S4.** (a) Optimized S_0_ geometry of EDOT-TPAOMe_4_ and the (c) HOMO and LUMO distributions in the corresponding geometries. (b) Corresponding root-mean-square deviations (RMSD) of EDOT-TPAOMe_4_.The structural changes between S_0_ (marked cyan) and S_1_ state (marked orange) is 1.0458Å. All TD-DFT calculations were conducted with Gaussian 16 and performed on the TD-M06-2X /6-31G(d,p) theoretical level.


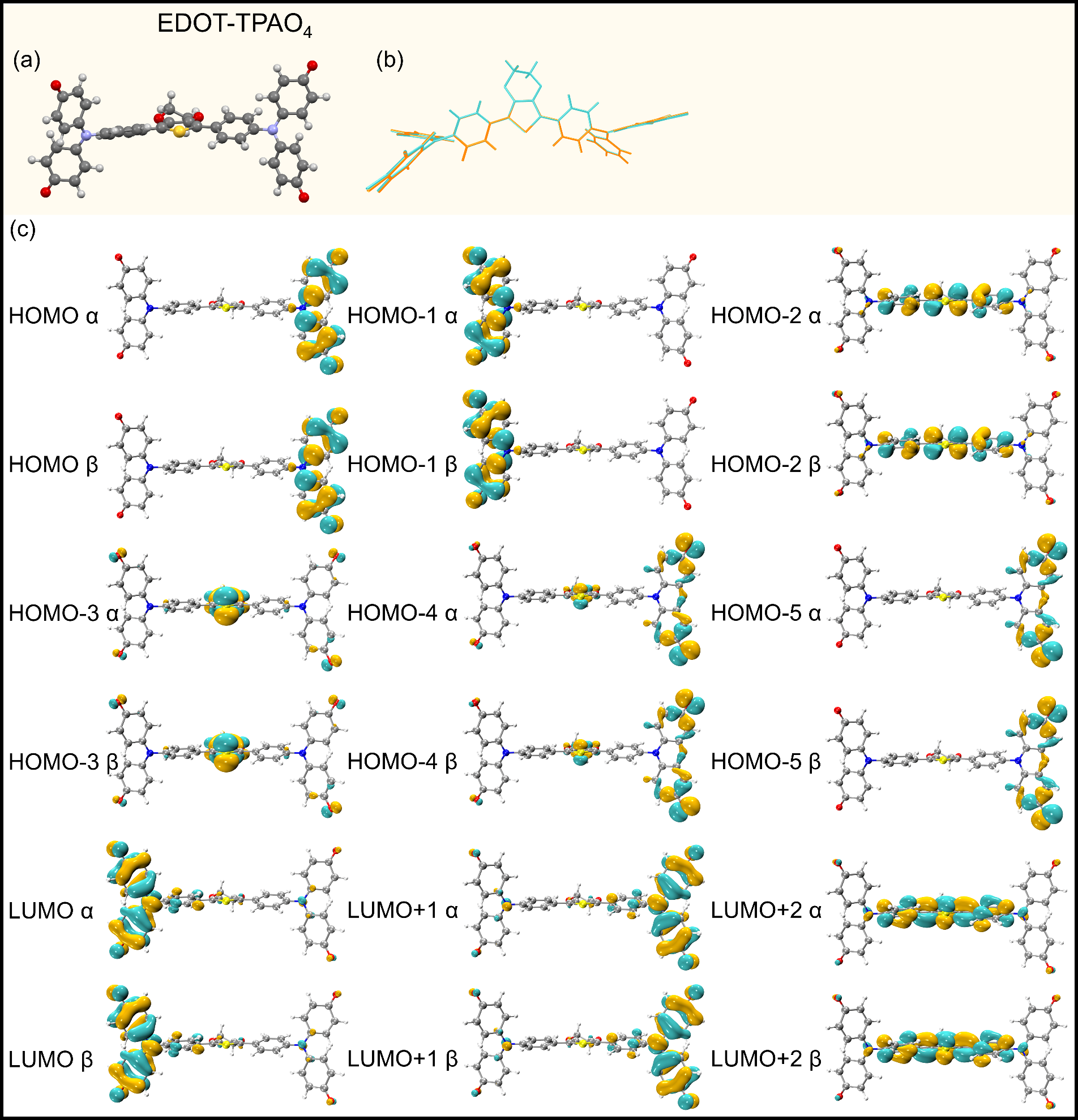


**Figure S5.** (a) Optimized S_0_ geometry of EDOT-TPAO_4_ and the (c) HOMO and LUMO distributions in the corresponding geometries. (b) Corresponding root-mean-square deviations (RMSD) of EDOT-TPAO_4_.The structural changes between S_0_ (marked cyan) and S_1_ state (marked orange) is 0.0378 Å. All TD-DFT calculations were conducted with Gaussian 16 and performed on the TD-UM06-2X /6-31G(d,p) theoretical level.


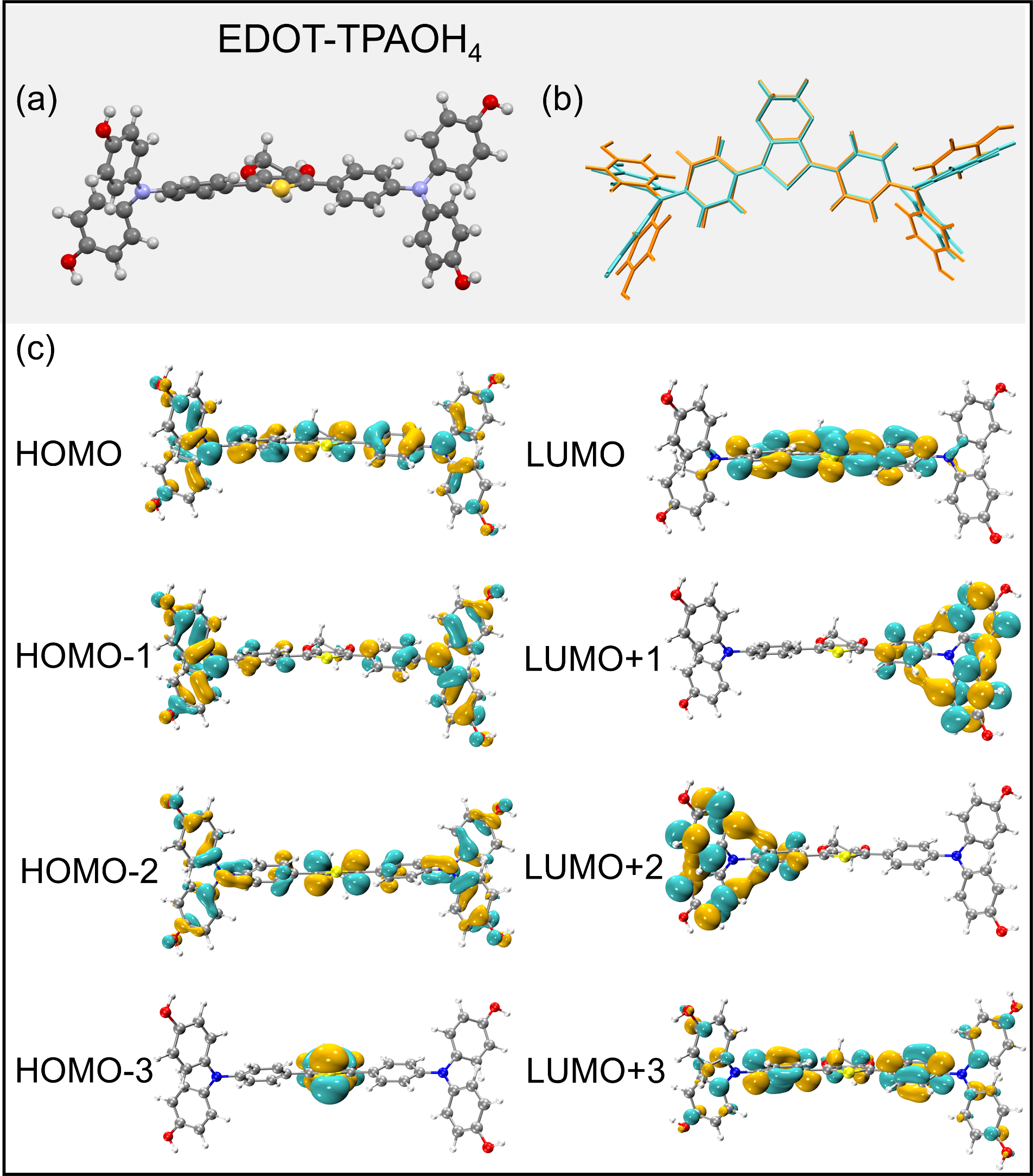


**Figure S6.** (a) Optimized S_0_ geometry of EDOT-TPAOH_4_ and the (c) HOMO and LUMO distributions in the corresponding geometries. (b) Corresponding root-mean-square deviations (RMSD) of EDOT-TPAOH_4_. The structural changes between S_0_ (marked cyan) and S_1_ state (marked orange) is 0.8883Å. All TD-DFT calculations were conducted with Gaussian 16 and performed on the TD-M06-2X /6-31G(d,p) theoretical level.


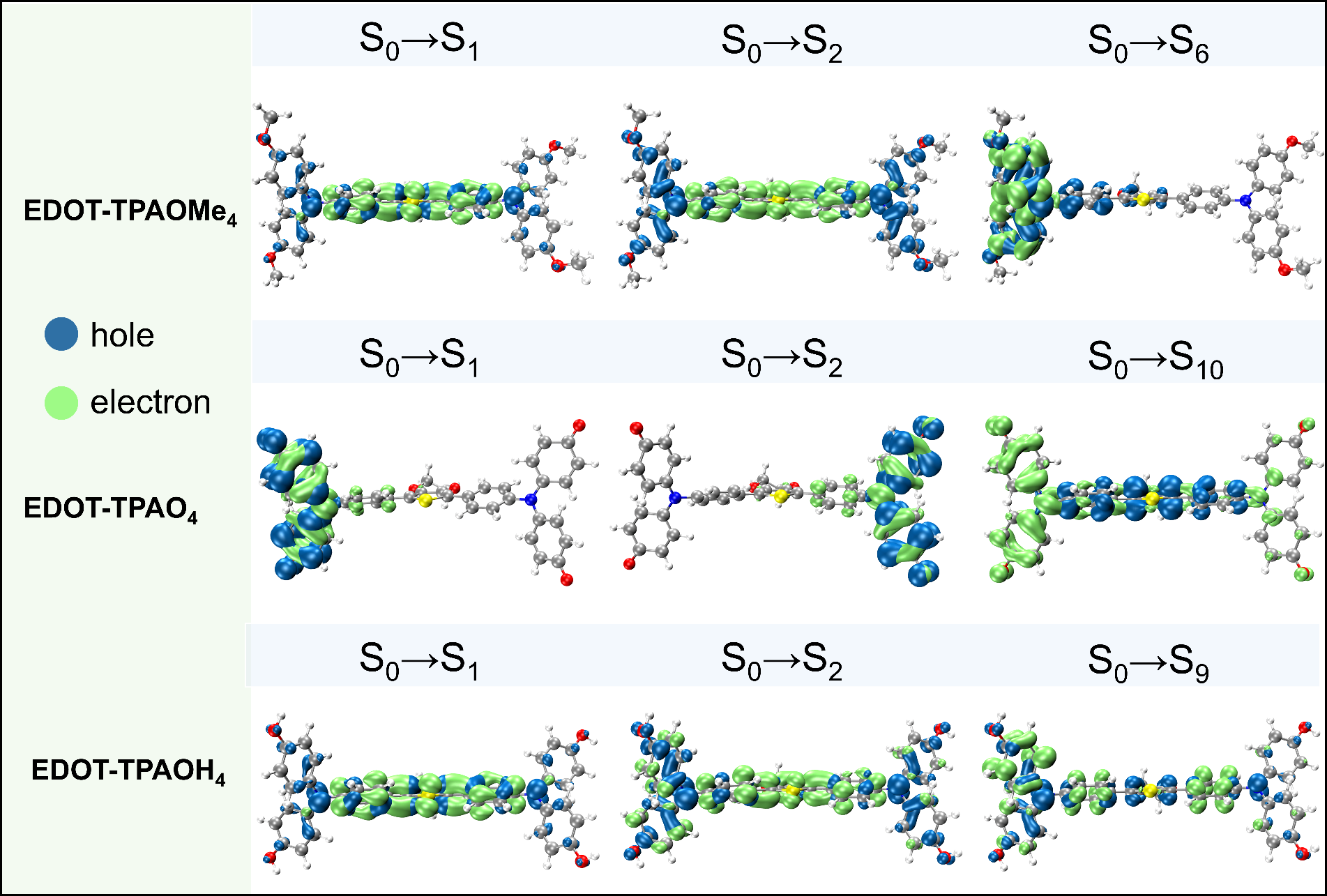


**Figure S7.** The hole (blue) and electron (green) distribution of major transitions in EDOT-TPAOMe_4_, EDOT-TPAO_4_ and EDOT-TPAOH_4_.


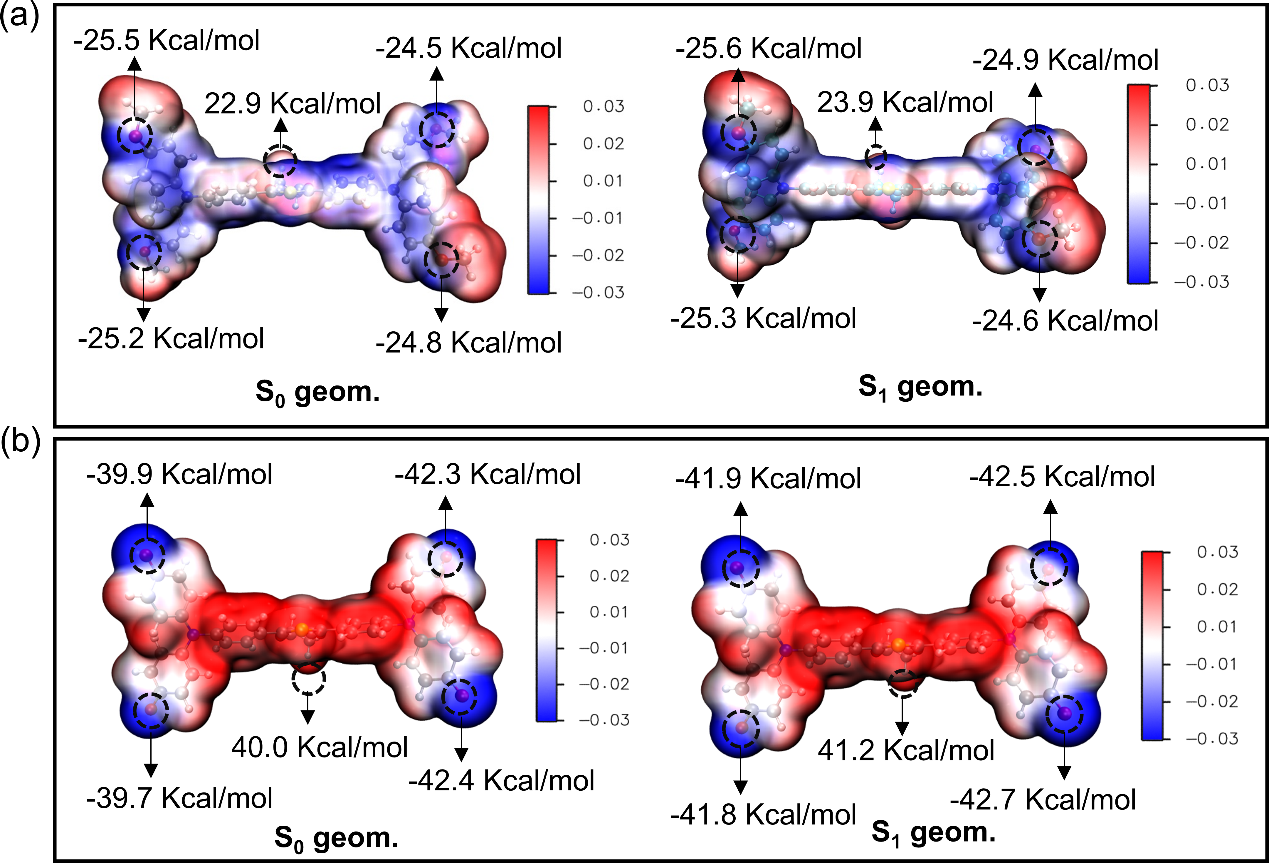


**Figure S8.** Electrostatic potential (ESP) maps of (a) **EDOT-TPAOMe_4_** and (b) **EDOT-TPAO_4_** in S_0_ and S_1_ geometry. Significant surface local minima and maxima of ESP are represented as blue and red spheres. Only the global minima and maxima labelled by black dashed circle, the unit is in kcal/mol.

**Table S3.** TD-DFT Calculated Electronic Excitation Properties of the **EDOT-TPA OMe_4_** and **EDOT-TPAO_4_** in the Optimized S_0_, S_1_, and T_1_ Geometries.

|  | **EDOT-TPAOMe_4_** | **EDOT-TPAO_4_** | |
| --- | --- | --- | --- |
| *E*_S0_ / eV | 0 | 0 |  |
| *E*_S1_ / eV | 3.425 | 2.150 |  |
| *E*_T1_ / eV | 2.559 | 0.166 |  |
| *E*_T2_ / eV | 3.232 | 0.297 |  |
| *E*_T1_ - *E*_S0_/ eV | 2.559 | 0.166 |  |

**Table S4**. Molecular orbital amplitude plots of HOMO and LUMO energy levels of **EDOT-TPA OMe_4,_ EDOT-TPAO_4_** **and** **EDOT-TPAOH_4_** at the (U)M06-2X / 6-31G (d, p) level by using the Gaussian 16 program package.

|  | Electronic | TD/(U)M06-2X/6-311G (d.p) | | |
| --- | --- | --- | --- | --- |
|  | transition | Energy / eV | f | Composition |
| **EDOT-TPAOMe_4_** | S_0_→S_1_ | 3.4252 eV 361.98 nm | 1.7480 | HOMO→LUMO  HOMO-1→LUMO |
|  | S_0_→S_2_ | 4.0577 eV 305.55 nm | 0.1199 | HOMO-1→LUMO  HOMO→LUMO+3 |
|  | S_0_→S_6_ | 4.5091 eV 274.96 nm | 0.2415 | HOMO→LUMO+5 |
|  |  |  |  | HOMO-1→LUMO+5 |
| **EDOT-TPAO_4_** | S_0_→S_1_ | 2.1504 eV 576.56 nm | 0.4919 | HOMO-1α→LUMO α  HOMO-1β-LUMO β |
|  | S_0_→S_2_ | 2.2363 eV 554.41 nm | 0.5999 | HOMOβ→LUMOβ+1  HOMOα→LUMOα+1 |
|  | S_0_→S_10_ | 2.9876 eV 415.00 nm | 1.1886 | HOMO-2 β→LUMOβ  HOMO-2 α→LUMOα |
| **EDOT-TPAOH_4_** | S_0_→S_1_ | 3.4317 eV 361.29 nm | 1.7017 | HOMO→LUMO  HOMO-1→LUMO |
|  | S_0_→S_2_ | 4.0719 eV 304.49 nm | 0.1144 | HOMO-1→LUMO  HOMO→LUMO+2 |
|  | S_0_→S_9_ | 4.6807 eV 264.88 nm | 0.2584 | HOMO→LUMO+8 |
|  |  |  |  | HOMO-1→LUMO+9 |

**
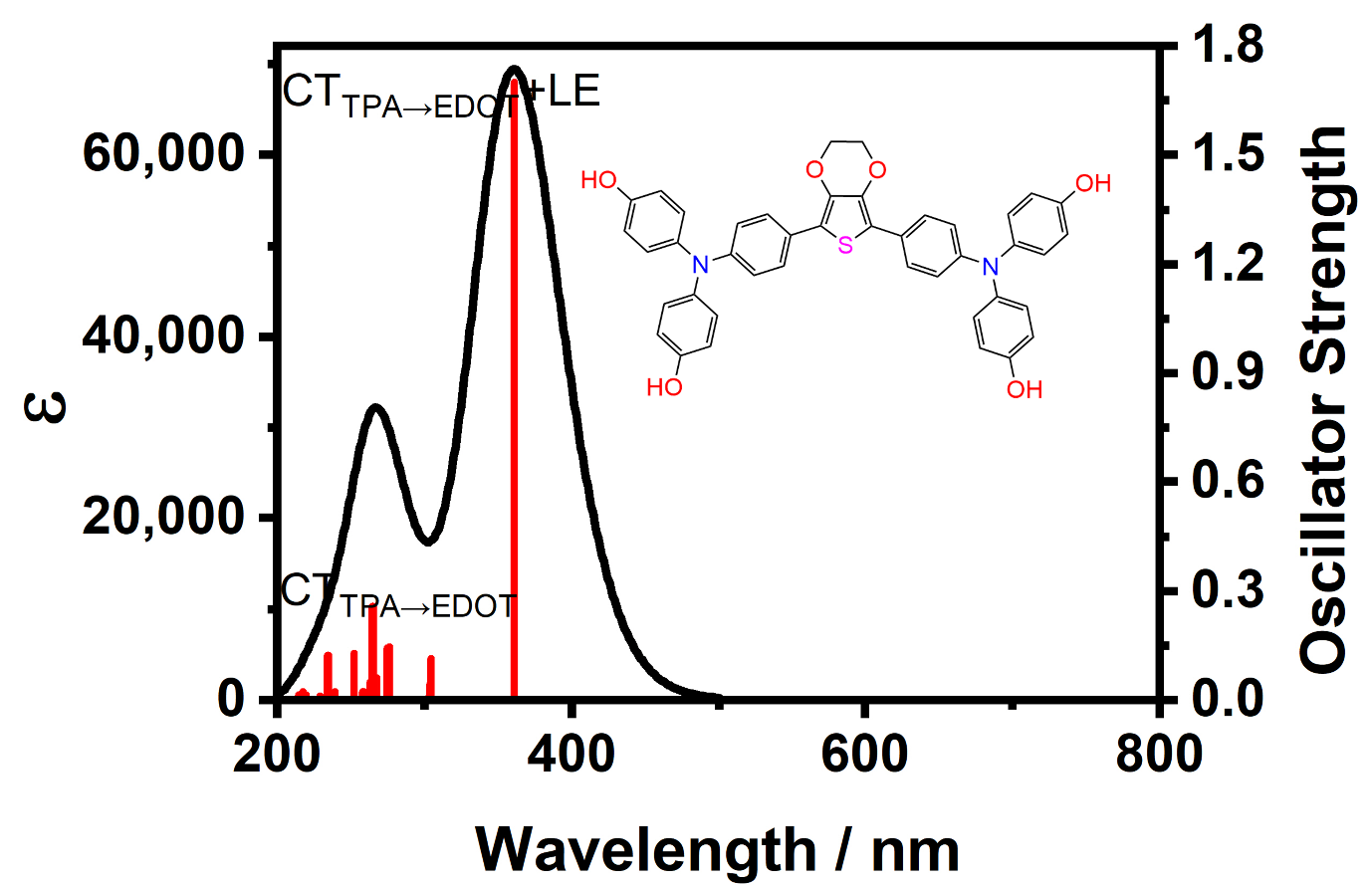
**

**Figure S9.** Simulated electronic absorption spectra of EDOT-TPAOH_4_ in optimized S_0_ geometries. The spectral profiles are reconstructed by the GaussView software based on excitation energies of the major transitions and the corresponding oscillator strength ( *f* ). The grey dashed line represents the measured steady-state UV−vis absorption with scaled intensity for comparison.

1. **Supplemental transient absorption spectra**


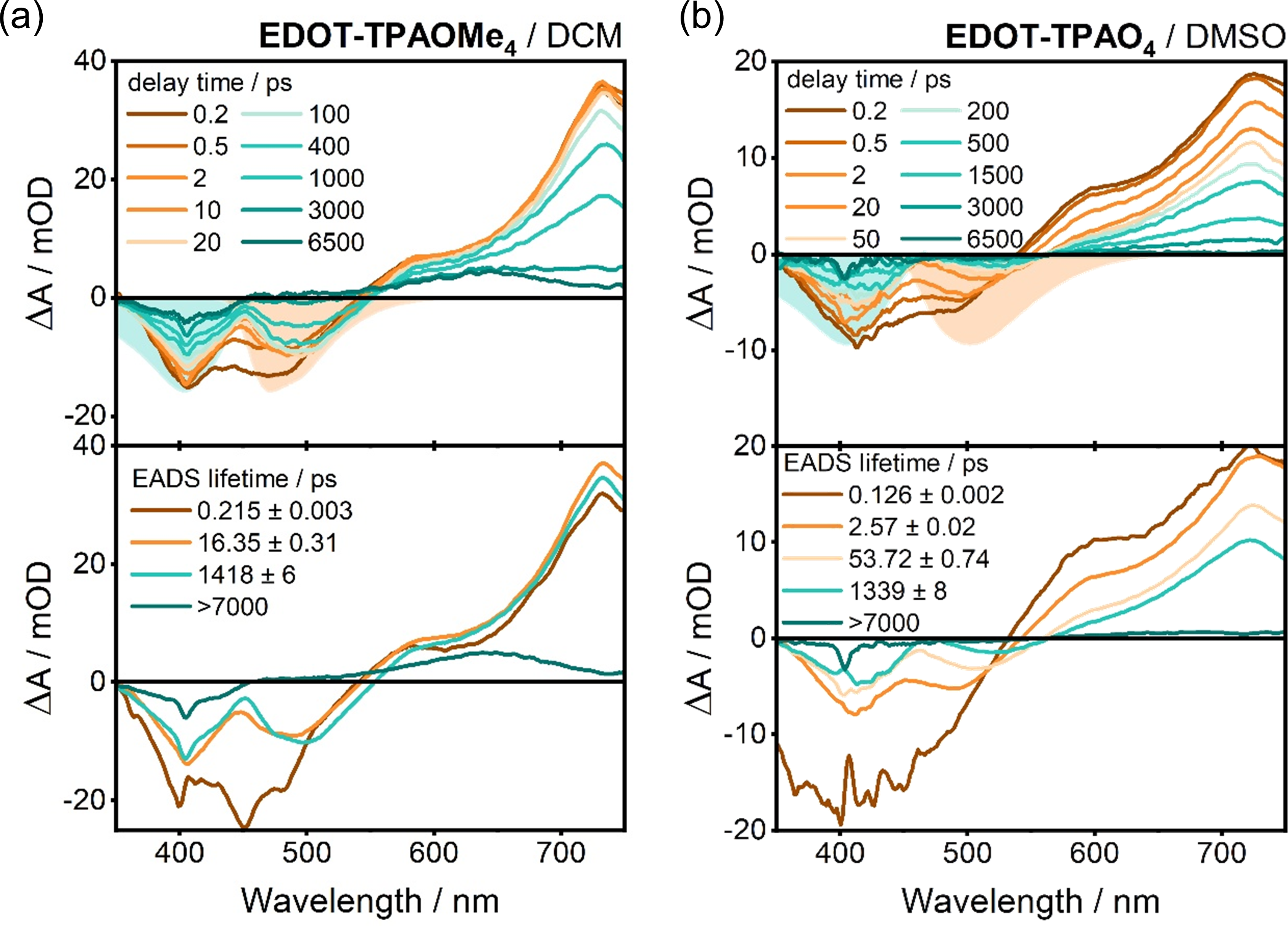


**Figure S10.** Time evolution of femtosecond TA spectra of (a) **EDOT-TPAOMe_4_** in DCM and (b) **EDOT-TPAO_4_** in DMSO upon excitation at 400 nm in the 8-ns time window. The cyan shaded areas represent the corresponding stationary absorption. Evolution-associated difference spectra (EADS) obtained from the global analysis based on a sequential model are shown in the lower panels below the corresponding TA spectra. The fitting time constants are shown in the legends.


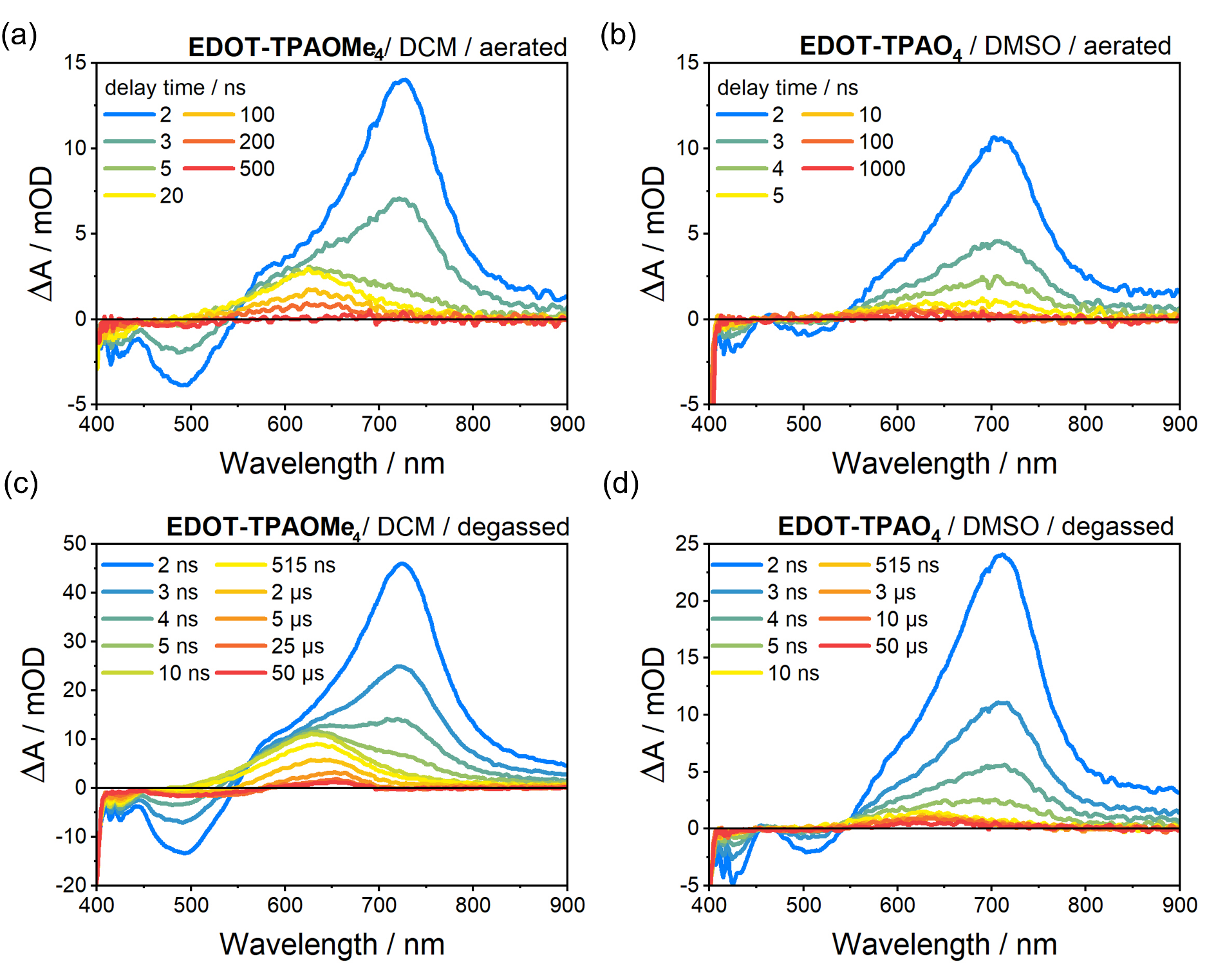


**Figure S11.** Time evolution of nanosecond TA spectra of **EDOT-TPAOMe_4_** in (a) aerated and (c) degassed DCM and **EDOT-TPAO_4_** in (b) aerated and (d) degassed DMSO upon excitation at 400 nm.

1. **Supplementary photothermal properties**

**
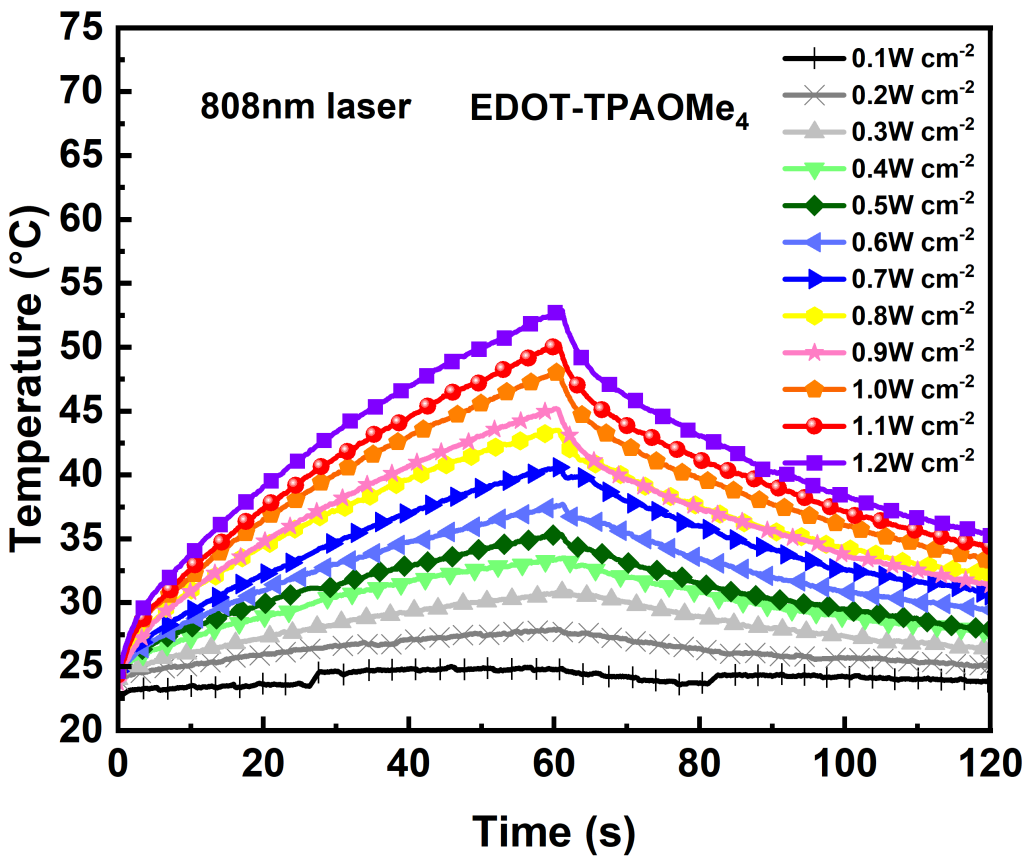
**

**Figure S12. EDOT-TPAOMe_4_** rising-cooling curves at a laser wavelength of 808nm and different power densities. (0.1-1.2 W cm^-2^).


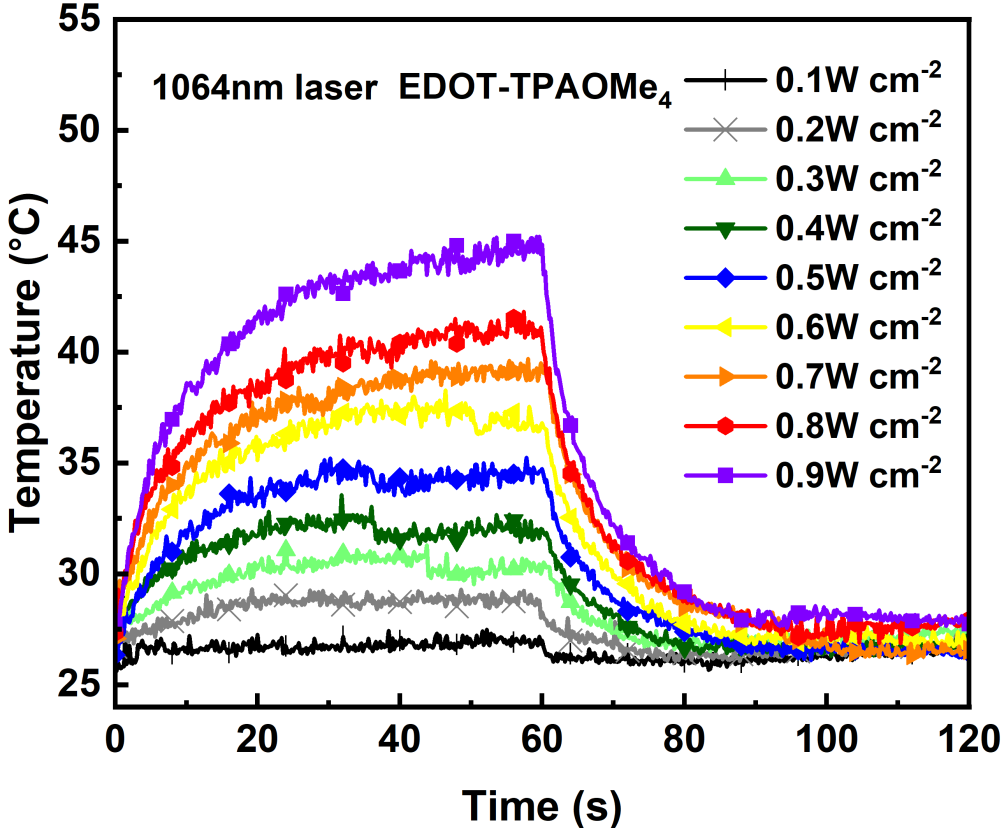


**Figure S13. EDOT-TPAOMe_4_** rising-cooling curves at a laser wavelength of 1064nm and different power densities. (0.1–0.9 W cm^-2^).


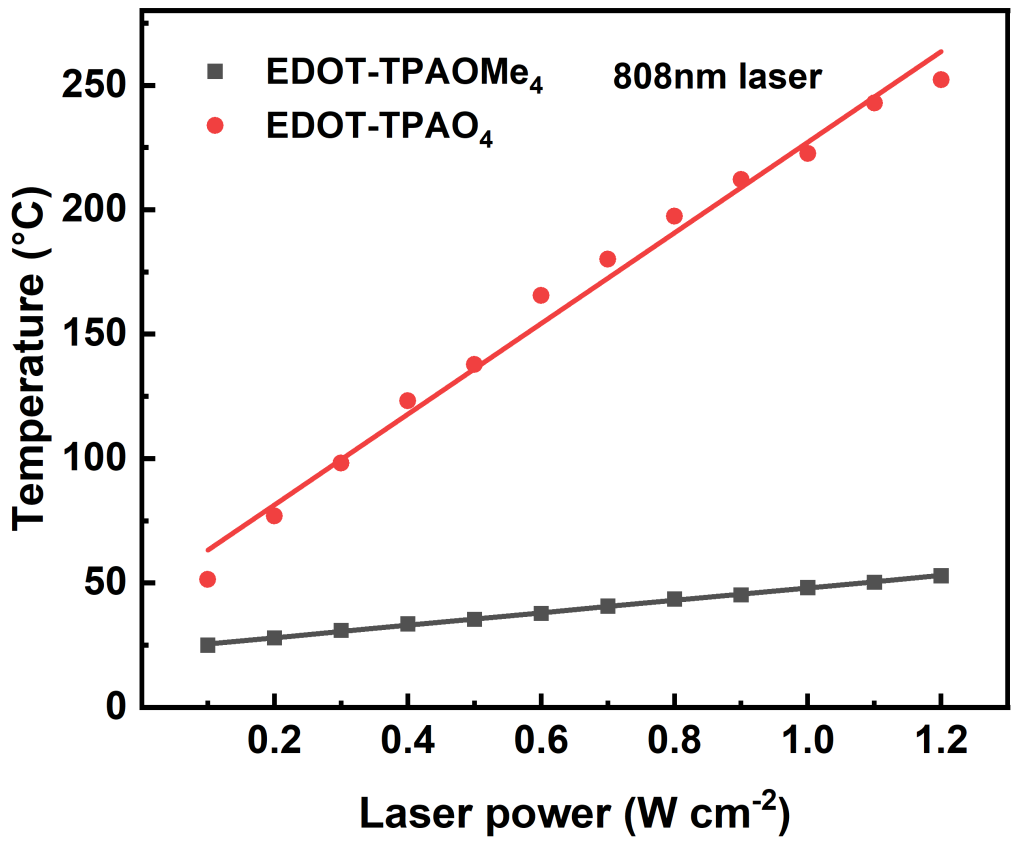


**Figure S14.** Fitting curves of maximum temperature and power density of **EDOT-TPAO_4_**/**EDOT-TPAOMe_4_** at 808 nm laser.


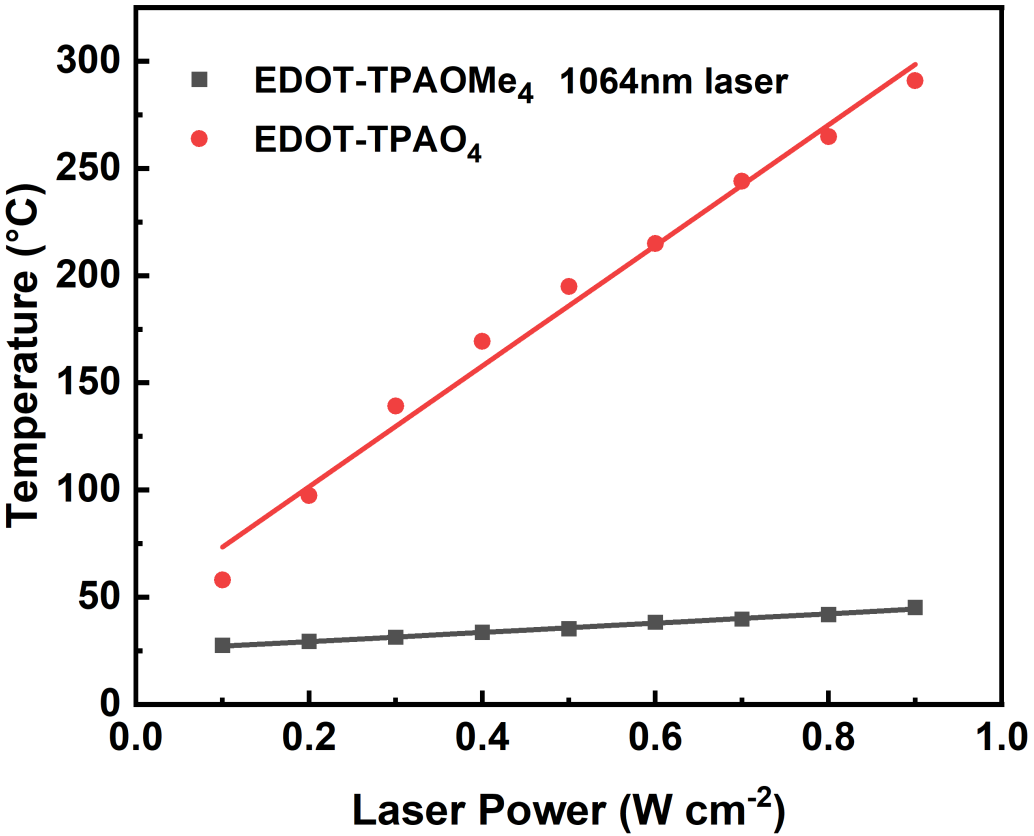


**Figure S15.** Fitting curves of maximum temperature and power density of **EDOT-TPAO_4_**/**EDOT-TPAOMe_4_** at 1064 nm laser.


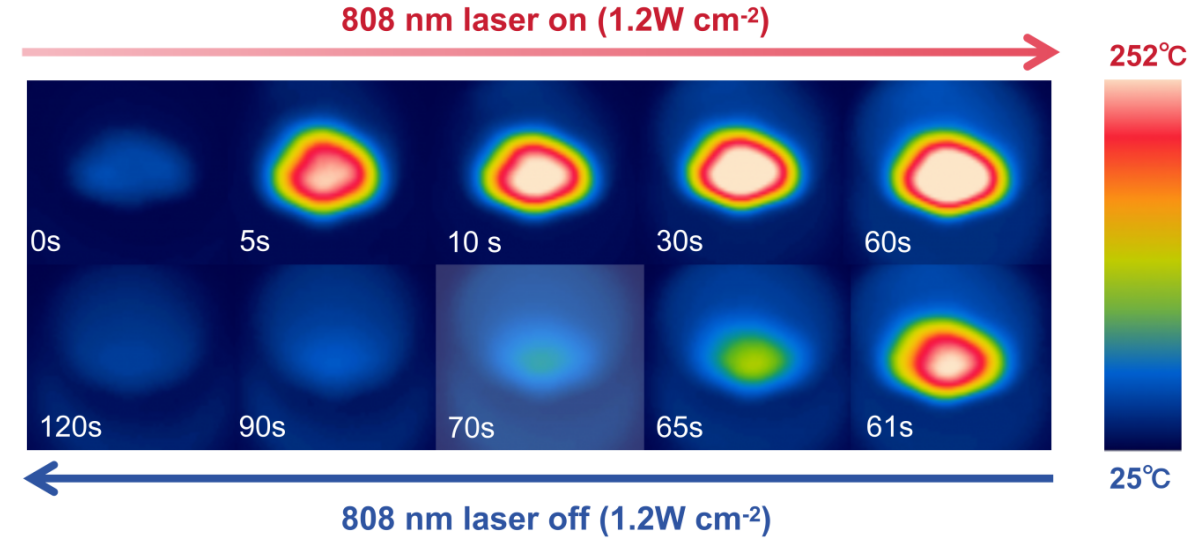


**Figure S16.** Infrared thermal images of **EDOT-TPAO_4_** powder under 808 nm laser irradiation (1.2 W cm^-2^).


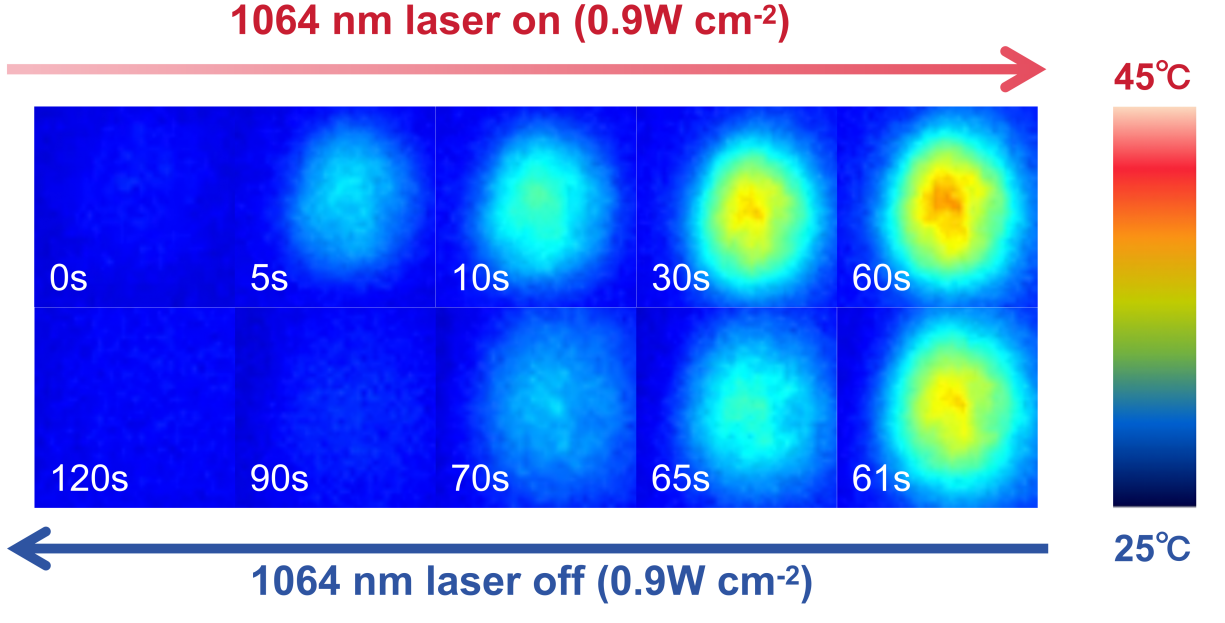


**Figure S17.** Infrared thermal images of **EDOT-TPAOMe_4_** powder under 1064nm laser irradiation (0.9 W cm^-2^).


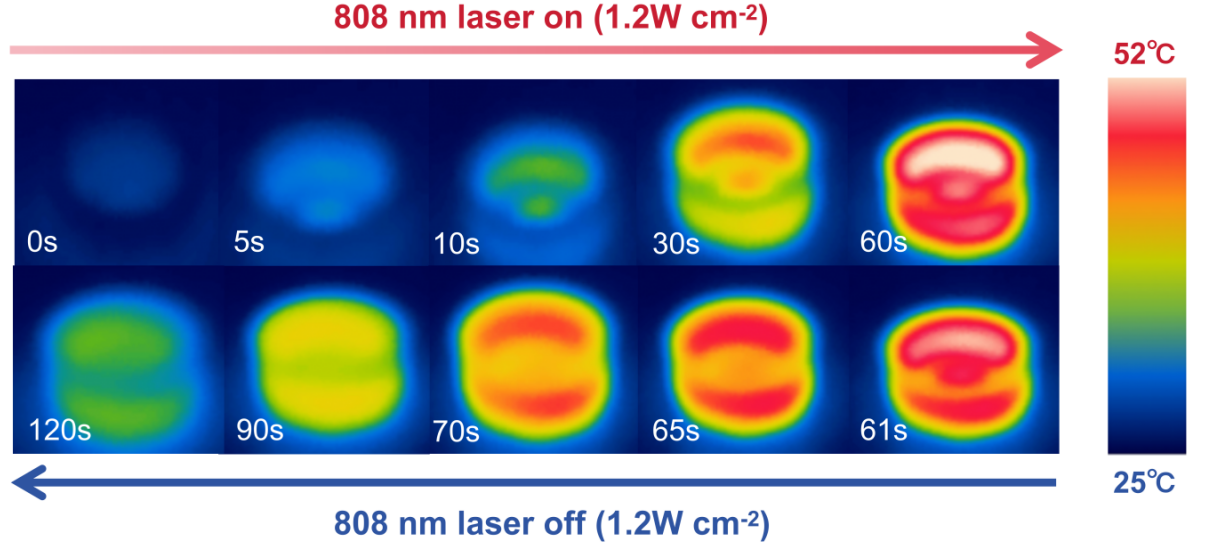


**Figure S18.** Infrared thermal images of **EDOT-TPAOMe_4_** powder under 808 nm laser irradiation (1.2 W cm^-2^).


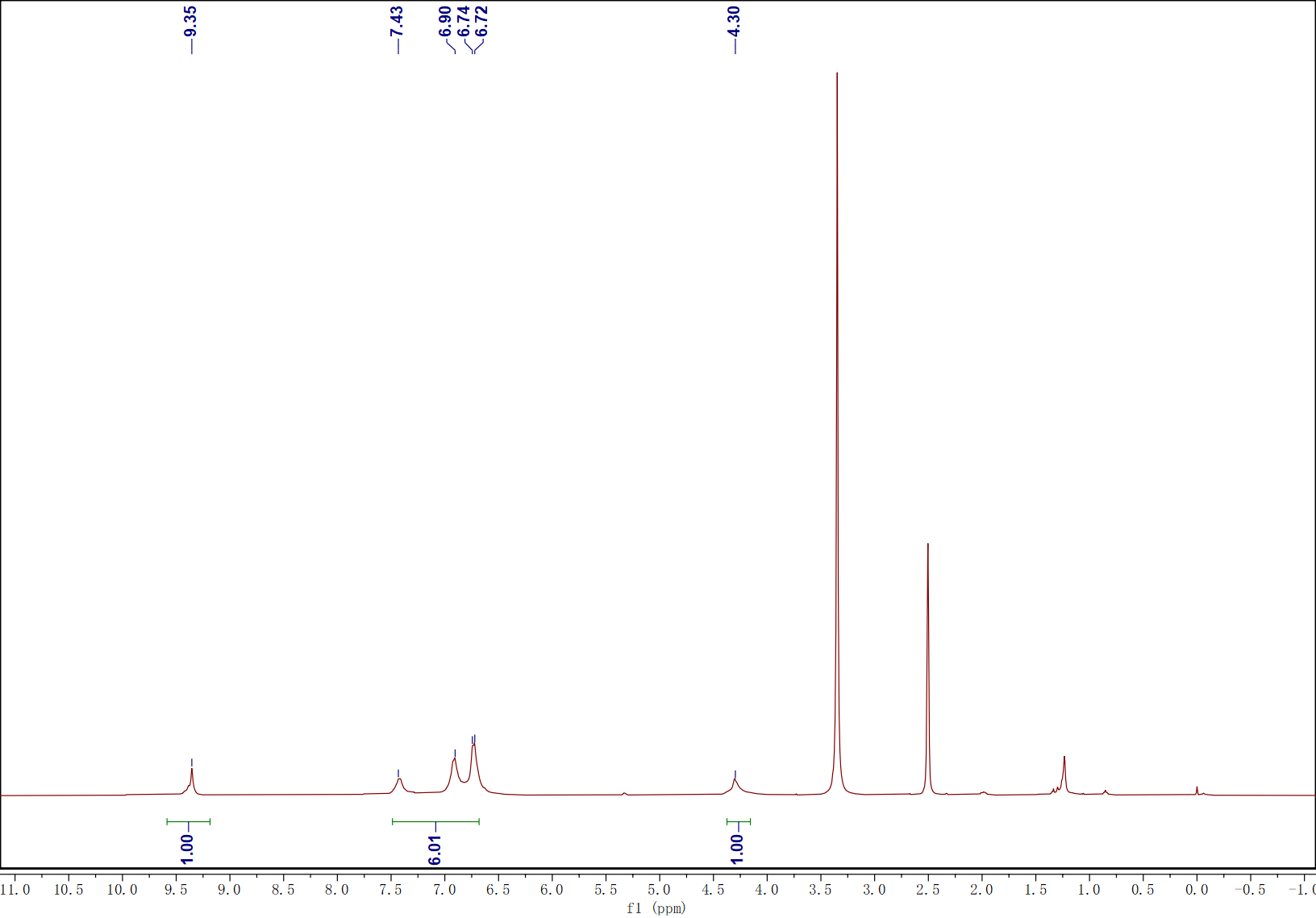


**Figure S19.** ^1^H-NMR of EDOT-TPAO_4_ after 10 photothermal cycles. (under 1064nm irradiation at 0.8 W cm^-2^)

1. **Characterization comparison of EDOT-TPAOMe_4_ and EDOT-TPAO_4_**

**Table S5.** *Eonset* represents the onset potential. HOMO and LUMO are determined from the oxidation and reduction onset, respectively. *E_g_^Opt^*: optical energy gap. *λ_abs_* is the wavelength of absorption edge in thin films. *T_max_*: Maximum photothermal conversion temperature.

| Compounds | *E*_onset_ (V) | *λ_abs,_*(nm) | *E*_g_^Opt^ (eV) | HOMO (eV) | LUMO (eV) | *T_max_* (℃) |
| --- | --- | --- | --- | --- | --- | --- |
| **EDOT-TPAOMe_4_** | 0.29 | 452 | 2.74 | -4.69 | -2.68 | 52 |
| **EDOT-TPAO_4_** | 0.35 | 985 | 1.26 | -4.75 | -3.49 | 290 |

1. **The powder absorption of organic small molecules**

**
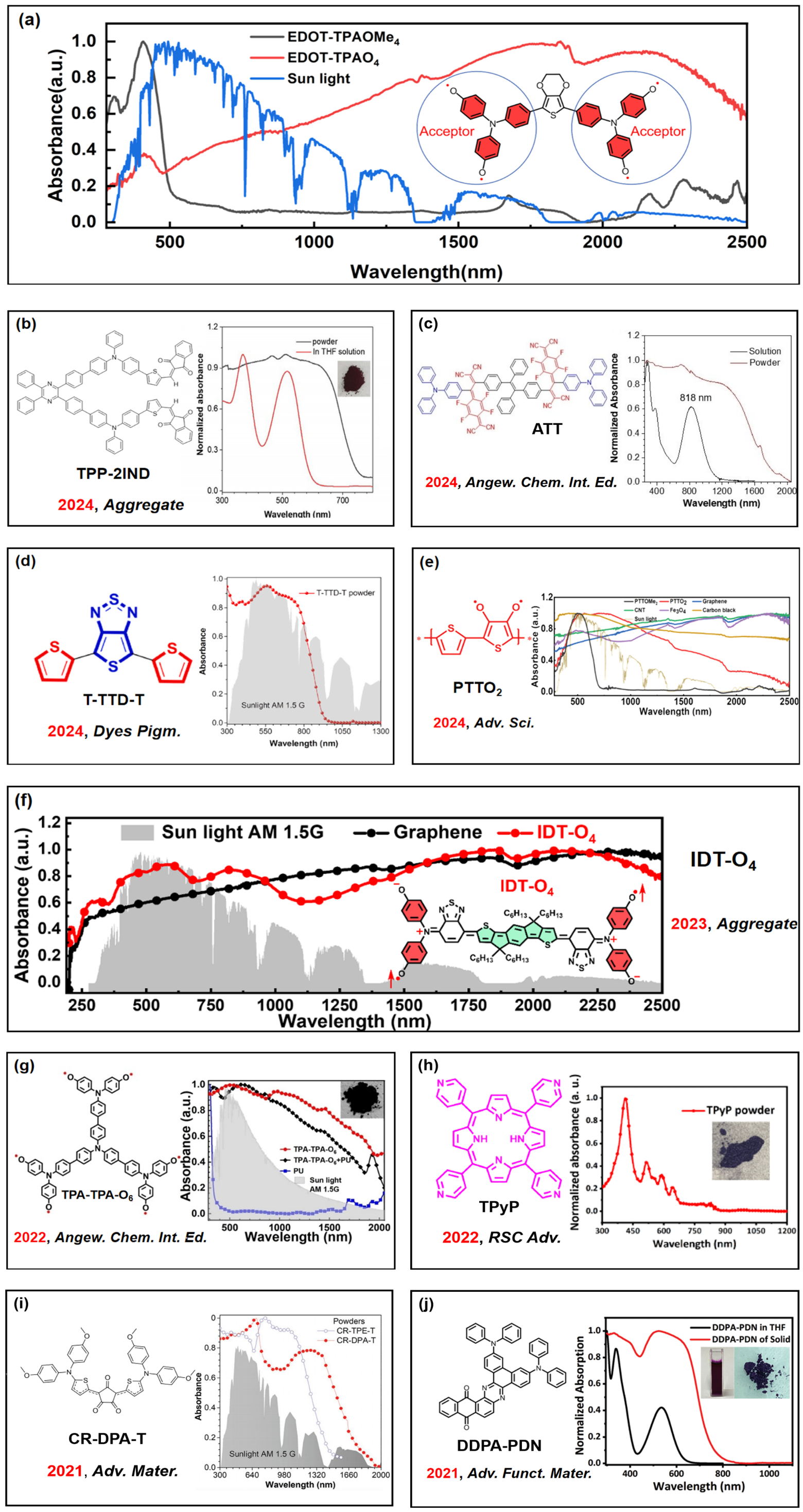
**

**Figure S20.** Comparison of powder absorption of **EDOT-TPAO_4_** with other small organic molecules. a, This work. b,TPP-2IND ^[13]^. c,ATT ^[14]^. d,T-TTD-T ^[15]^. e, PPTO_2_ ^[16]^. f,IDT-O_4_(our previous work) ^[12]^. g,TPA-TPA-O_6_(our previous work) ^[10]^. h,TPyP ^[17]^. i,CR-DPA-T ^[18]^. j,DDPA-PDN ^[19]^.

## The calculation of the efficiency for solar to vapor generation

**Table S6.** Detail calculation method of the water evaporation rate/solar-driven water evaporation conversion efficiency

| The conversion efficiency *η* of solar energy in photothermal assisted water evaporation was calculated as the following formula： | *η* = *ṁ h_LV_* /*Copt P_0_* |
| --- | --- |
| Where *ṁ* refers to the mass flux (evaporation rate) of water, *h_LV_* refers to the total liquid-vapor phase-change enthalpy. *Q* is the energy provided to heat the system from the initial temperature *T_0_* to a final temperature *T*, *△h_vap_* is the latent heat of vaporization of water, *P_0_* is the nominal solar irradiation value of 1 kW m^-2^, and *Copt* represents the optical concentration (*Copt* = 1). | *Q* = *C_liquid_* × (*T* - *T_0_*)  *Δh_vap_* = *Q_1_* + *Δh_100_* + *Q_2_*  *h_LV_* = *Q* + *Δh_vap_*  *Q_1_* = *C*_liquid_ × (100 - *T*)  *Q_2_* = *C*_vapor_ × (*T* - 100) |
| In this study, *C_liquid_*, the specific heat capacity of liquid water is a constant of 4.18J/g℃. *C_vapor_*, the specific heat capacity of water vaper is a constant of 1.865 J/g℃. *△h_100_* is the latent heat of vaporization of water at 100℃, taken to be 2260 kJ/kg. | |

**Detailed calculation of the EDOT-TPAO_4_**

*Q* = *C_liquid_* × (*T* - *T_0_*) = 4.18×(46.4075 - 30.6379) = 65.9169 kJ kg^-1^

*Q_1_* = *C_liquid_* × (100 - *T*) = 4.18×(100 - 46.4075) = 224.0166 kJ kg^-1^

*Q_2_* = *C_vapor_* × (*T* - 100) = 1.865×(46.4075 - 100) = -99.9500 kJ kg^-1^

*Δh_vap_* = *Q_1_* + *Δh_100_* + *Q_2_* = 224.0166 + 2260 - 99.9500 = 2384.0666 kJ kg^-1^

*h_LV_* = *Q* + *Δh_vap_* = 65.9169 + 2384.0666 = 2449.9835 kJ kg^-1^

*η* = *ṁ h_LV_* /*Copt P_0_* =1.433 × 2449.9835 / 1 × 1 = 97.5 %

## Comparison of photothermal properties of different materials

**Table S7.** The maximum temperature comparison of various photothermal functional materials under different laser power and laser wavelength.

| **Materials** | **Laser Power**  **（W/cm^-2^）** | **Laser**  **Wavelength(nm)** | **Maximum**  **Temperature(℃)** | **Ref.** |
| --- | --- | --- | --- | --- |
| EDOT-TPAO_4_ | 0.9 | 1064 | 290.0 | This work |
| EDOT-TPAO_4_ | 1.2 | 808 | 252.2 | This work |
| PTTO_2_ | 1.2 | 808 | 273 | ^[16]^ |
| CR-DPA-T | 0.8 | 808 | 110.0 | ^[18]^ |
| Zr-PDI | 0.7 | 808 | 114.0 | ^[20]^ |
| MNC | 0.8 | 808 | 163.8 | ^[21]^ |
| CR-TPE-T | 1.2 | 808 | 129.0 | ^[22]^ |
| IDT-O_4_ | 0.9 | 808 | 248.6 | ^[12]^ |
| Th_2_-O_4_ | 0.8 | 808 | 216.0 | ^[12]^ |
| Th-O_4_ | 0.8 | 808 | 190.0 | ^[12]^ |
| NDI-TPA-O_4_ | 1.0 | 808 | 227.9 | ^[12]^ |
| DPA-BT-O_4_ | 0.8 | 808 | 95.0 | ^[12]^ |
| TPA-TPA-O_6_ | 0.8 | 808 | 216.0 | ^[10]^ |
| GT-COF-3 | 0.8 | 808 | 225.0 | ^[23]^ |
| DTC | 0.7 | 808 | 71.3 | ^[24]^ |
| NKU-123 | 1.0 | 808 | 215.1 | ^[25]^ |
| ATT | 1.0 | 808 | 250 | ^[14]^ |
| DDPA-PDN | 0.9 | 655 | 201 | ^[19]^ |
| TPP-2IND | 0.9 | 660 | 200 | ^[13]^ |
| Tpyp | 1.0 | 730 | 156 | ^[17]^ |
| NSM1 | 1.0 | 808 | 143 | ^[26]^ |

|  |
| --- |

**Table S8.** Summary of previously reported absorption spectra of photothermal materials and partial water evaporation data

| Materials | Chemical structure | Water evaporation | Solid/Solution absorption range | Ref |
| --- | --- | --- | --- | --- |
| EDOT-TPAO_4_ | 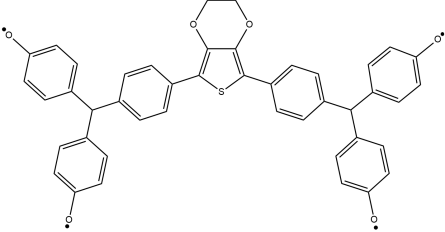 | 97.5% | 300-2500 nm  (solid) | This work |
| PTTO_2_ | 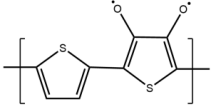 | 83.3% | 300~2000 nm  (solid) | ^[16]^ |
| CR-DPA-T | 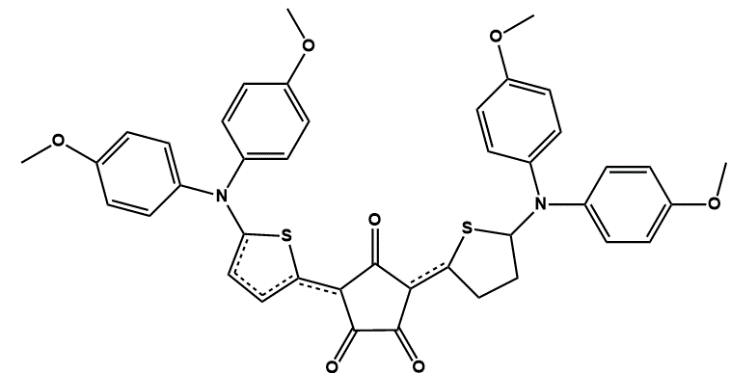 | None | 300~835 nm  (solid) | ^[18]^ |
| Zr-PDI | 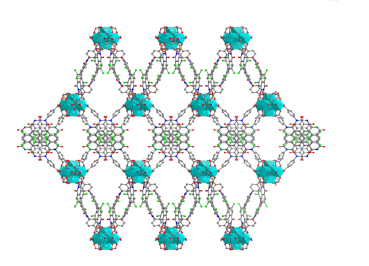 | None | 250~900 nm  (solid) | ^[20]^ |
| CR-TPE-T | 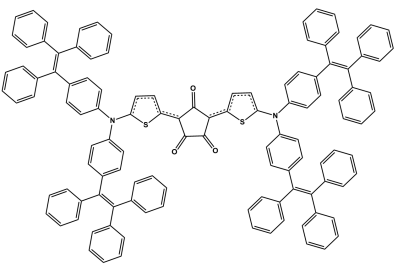 | 87.2% | 300~1600nm  (solid) | ^[22]^ |
| IDT-O_4_ | 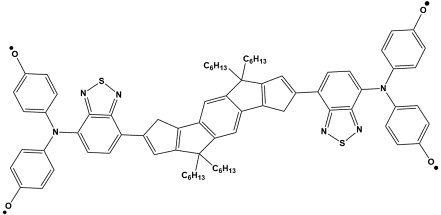 | 94.38% | 300~2500 nm  (solid) | ^[12]^ |
| TPA-TPA-O_6_ | 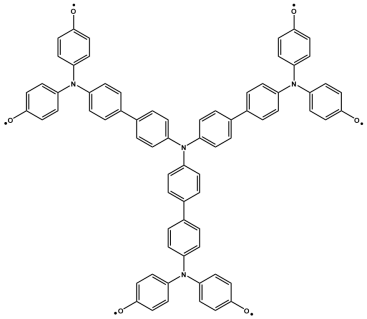 | 89.41% | 400~825nm  (solid) | ^[10]^ |
| GT-COF-3 | 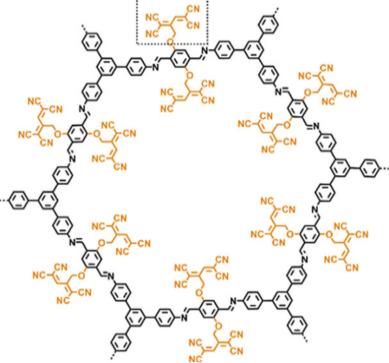 | 90.7% | 250~1400nm  (solid) | ^[23]^ |
| DTC | 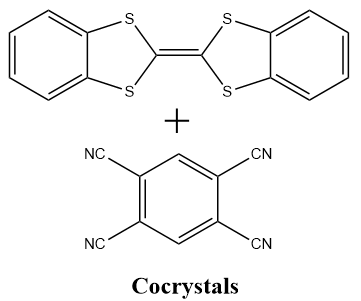 | None | 300~1000nm  (solid) | ^[24]^ |
| ATT | 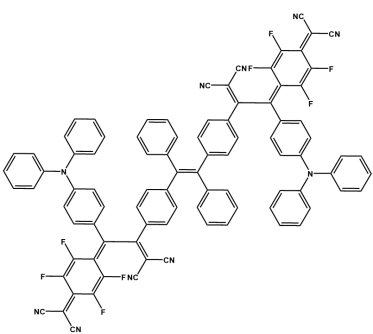 | None | 200~800nm  (solid) | ^[14]^ |
| DDPA-PDN | 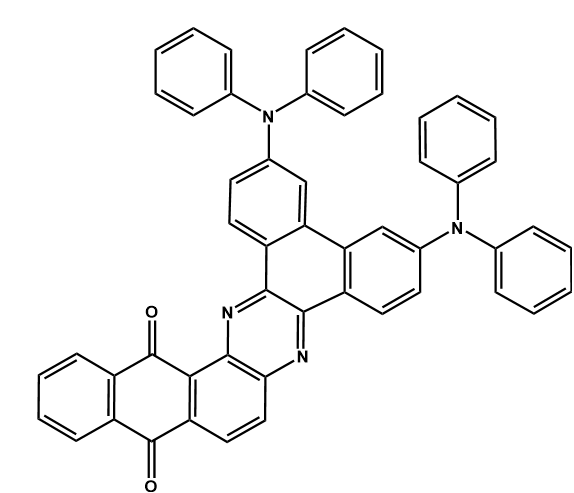 | 73.98% | 300~880nm  (solid) | ^[19]^ |
| TPP-2IND | 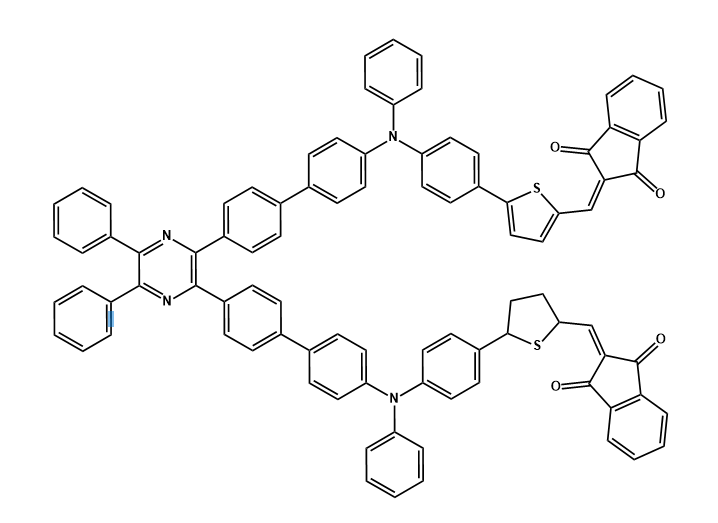 | 65.8% | 300~780nm  (solid) | ^[13]^ |
| Tpyp | 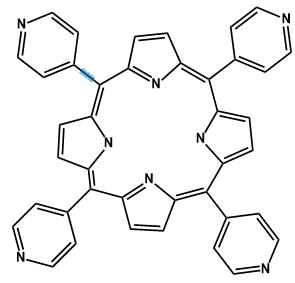 | 56% | 300~850nm  (solid) | ^[17]^ |
| NSM1 | 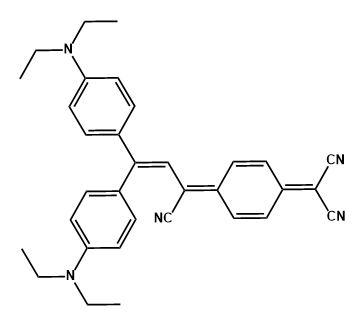 | None | 300~1100nm  (solution) | ^[26]^ |

##

## Comparison of water evaporation properties of different materials

**Table S9.** The water evaporation rate and water evaporation conversion efficiency of different photothermal functional materials under 1 sun irradiation (1 kW m^-2^).

| **system** | **Materials** | **Evaporation**  **Conversion**  **Efficiency(%)** | **Evaporation rate**  **(kg m^-2^ h^-1^)** | **Ref.** |
| --- | --- | --- | --- | --- |
| **Organic small molecule** | EDOT-TPAO_4_ | 97.5 | 1.433 | This wok |
|  | IDT-O_4_ | 94.38 | 1.365 | ^[12]^ |
|  | DDPA-PDN | 73.98 | 0.890 | ^[19]^ |
|  | CR-TPE-T | 87.20 | 1.272 | ^[22]^ |
|  | AFQ | 57.00 | 1.330 | ^[27]^ |
|  | 4OCSPC | 86.60 | 1.262 | ^[28]^ |
|  | P(DPP)_4_ | 90.60 | 1.304 | ^[29]^ |
|  | GDPA-QCN | 90.40 | 1.300 | ^[30]^ |
|  | DPP-INCN | 69.10 | 0.770 | ^[31]^ |
|  | TPyP | 56.00 | 0.810 | ^[17]^ |
|  | TPA-TPA-O_6_ | 89.41 | 1.293 | ^[10]^ |
|  | TTP-2IND | 65.8 | 1.04 | ^[13]^ |
|  | T-TTD-T | 85.2 | 1.2366 | ^[15]^ |
|  | 2TP-BBT | 94.3 | 1.35 | ^[32]^ |
| **Organic polymer** | GT-COF-3 | 90.70 | 1.314 | ^[23]^ |
|  | GS-COF-2-7 d | 92.00 | 1.340 | ^[33]^ |
|  | BHMS | 84.70 | 1.390 | ^[34]^ |
|  | PPy/SS | 58.00 | 0.920 | ^[35]^ |
|  | PPy-wood | 72.50 | 1.014 | ^[36]^ |
|  | Polymer film | 80.50 | 1.169 | ^[37]^ |
|  | PDA-CA | 86.00 | 1.360 | ^[38]^ |
|  | PDA-wood | 87.00 | 1.380 | ^[39]^ |
|  | Nylon-C | 83.10 | 1.240 | ^[40]^ |
| **Inorganic** | CNT/CNC | 87.40 | 1.350 | ^[41]^ |
|  | HPCM-PHS | 90.80 | 1.380 | ^[42]^ |
|  | Ti_3_C_2_ gel | 93.70 | 1.370 | ^[43]^ |
|  | MDP/SS | 84.30 | 1.222 | ^[44]^ |
|  | HKUST-1/CNF | 84.30 | 1.330 | ^[45]^ |
|  | rGO/PU | 65.00 | 0.900 | ^[46]^ |
|  | Carbon fiber | 80.00 | 1.300 | ^[47]^ |
|  | Ti3C2 MXene | 71.00 | 1.310 | ^[48]^ |
|  | DLS | 65.00 | 1.100 | ^[49]^ |

**Reference**

1. H. Li, K. Fu, A. Hagfeldt, M. Grätzel, S. G. Mhaisalkar, A. C. Grimsdale, *Angew. Chem. Int. Ed.* **2014**, *53*, 4085-4088.

2. S. van Buuren, K. Groothuis-Oudshoorn, *J. Stat. Softw* **2011**, *45*, 1 - 67.

3. I. H. M. van Stokkum, D. S. Larsen, R. van Grondelle, *Biochimica Et Biophysica Acta-Bioenergetics* **2004**, *1657*, 82-104.

4. M. J. T. Frisch, G. W.; Schlegel, H. B.; Scuseria, G. E.; Robb, M. A.; Cheeseman, J. R.; Scalmani, G.; Barone, V.; Petersson, G. A.; Nakatsuji, H.; Li, X.; Caricato, M.; Marenich, A. V.; Bloino, J.; Janesko, B. G.; Gomperts, R.; Mennucci, B.; Hratchian, H. P.; Ortiz, J. V.; Izmaylov, A. F.; Sonnenberg, J. L.; Williams; Ding, F.; Lipparini, F.; Egidi, F.; Goings, J.; Peng, B.; Petrone, A.; Henderson, T.; Ranasinghe, D.; Zakrzewski, V. G.; Gao, J.; Rega, N.; Zheng, G.; Liang, W.; Hada, M.; Ehara, M.; Toyota, K.; Fukuda, R.; Hasegawa, J.; Ishida, M.; Nakajima, T.; Honda, Y.; Kitao, O.; Nakai, H.; Vreven, T.; Throssell, K.; Montgomery Jr., J. A.; Peralta, J. E.; Ogliaro, F.; Bearpark, M. J.; Heyd, J. J.; Brothers, E. N.; Kudin, K. N.; Staroverov, V. N.; Keith, T. A.; Kobayashi, R.; Normand, J.; Raghavachari, K.; Rendell, A. P.; Burant, J. C.; Iyengar, S. S.; Tomasi, J.; Cossi, M.; Millam, J. M.; Klene, M.; Adamo, C.; Cammi, R.; Ochterski, J. W.; Martin, R. L.; Morokuma, K.; Farkas, O.; Foresman, J. B.; Fox, D. J. , **2016**.

5. Y. Zhao, D. G. Truhlar, *Theor. Chem. Acc.* **2008**, *120*, 215-241.

6. W. J. Hehre, R. Ditchfield, J. A. Pople, *Chem. Phys* **1972**, *56*, 2257-2261.

7. J. Zhang, T. Lu, *Phys. Chem. Chem. Phys.* **2021**, *23*, 20323-20328.

8. T. Lu, F. Chen, *J. Comput. Chem.* **2012**, *33*, 580-592.

9. E. R. Johnson, S. Keinan, P. Mori-Sánchez, J. Contreras-García, A. J. Cohen, W. Yang, *J.Am.Chem.Soc.* **2010**, *132*, 6498-6506.

10. Z. Wang, J. Zhou, Y. Zhang, W. Zhu, Y. Li, *Angew. Chem., Int. Ed.* **2022**, *61*, e202113653.

11. P. Yang, T. Y. Wang, J. H. Zhang, H. J. Zhang, W. J. Bai, G. G. Duan, W. Zhang, J. R. Wu, Z. P. Gu, Y. W. Li, *Sci. China:Chem.* **2023**, *66*, 1520-1528.

12. J. Huang, Z. Wang, W. Zhu, Y. Li, *Aggregate* **2024**, *5*, e426.

13. J.-C. Yang, L. Wu, L. Wang, R. Ren, P. Chen, C. Qi, H.-T. Feng, B. Z. Tang, *Aggregate* **2024**, *5*, e535.

14. P. Han, H. Xu, G. Zhang, A. Qin, B. Z. Tang, *Angew. Chem., Int. Ed.* **2024**, *63*, e202406381.

15. L. X. Guo, X. W. Kong, R. D. Li, J. M. Sun, G. Wang, X. G. Gu, *Dyes Pigm.* **2024**, *227*, 112195.

16. Q. Wei, J. Huang, Q. Meng, Z. Zhang, S. Gu, Y. Li, *Adv Sci* **2024**, *11*, 2406800.

17. Y. Zhang, H. Yan, X. Wang, Z. Zhang, F. Liu, S. Tu, X. Chen, *RSC Adv* **2022**, *12*, 28997-29002.

18. J. Sun, E. Zhao, J. Liang, H. Li, S. Zhao, G. Wang, X. Gu, B. Z. Tang, *Adv. Mater.* **2022**, *34*, 2108048.

19. Y. Cui, J. Liu, Z. Li, M. Ji, M. Zhao, M. Shen, X. Han, T. Jia, C. Li, Y. Wang, *Adv. Funct. Mater.* **2021**, *31*, 2106247.

20. B. Lü, Y. Chen, P. Li, B. Wang, K. Müllen, M. Yin, *Nat. Commun.* **2019**, *10*, 767.

21. H. Zou, X. Meng, X. Zhao, J. Qiu, *Adv. Mater.* **2023**, *35*, 2207262.

22. G. Chen, J. Sun, Q. Peng, Q. Sun, G. Wang, Y. Cai, X. Gu, Z. Shuai, B. Z. Tang, *Adv. Mater.* **2020**, *32*, e1908537.

23. X. Tang, Z. Chen, Q. Xu, Y. Su, H. Xu, S. Horike, H. Zhang, Y. Li, C. Gu, *CCS Chemistry* **2022**, *4*, 2842-2853.

24. Y. Wang, W. G. Zhu, W. N. Du, X. F. Liu, X. T. Zhang, H. L. Dong, W. P. Hu, *Angew. Chem., Int. Ed.* **2018**, *57*, 3963-3967.

25. W. Lan, X. Gou, Y. Wu, N. Liu, L. Lu, P. Cheng, W. Shi, *Angew. Chem. Int. Ed.* **2024**, *63*, e202401766.

26. X. Li, D. Zhang, G. Lu, T. He, Y. Wan, M.-K. Tse, C. Ren, P. Wang, S. Li, J. Luo, C.-S. Lee, *Adv. Mater.* **2021**, *33*, 2102799.

27. Y.-T. Chen, X. Wen, J. He, Z. Li, S. Zhu, W. Chen, J. Yu, Y. Guo, S. Ni, S. Chen, L. Dang, M.-D. Li, *ACS Appl. Mater. Interfaces* **2022**, *14*, 28781-28791.

28. X. Han, Z. Y. Wang, M. H. Shen, J. Liu, Y. X. Lei, Z. Q. Li, T. Jia, Y. Wang, *J. Mater. Chem. A* **2021**, *9*, 24452-24459.

29. X. Zhang, Y. Li, Z. Chen, P. Li, R. Chen, X. Peng, *Dyes Pigm.* **2021**, *192*, 109460.

30. J. Liu, Y. Cui, Y. Pan, Z. Chen, T. Jia, C. Li, Y. Wang, *Angew. Chem. Int. Ed.* **2022**, *61*, e202117087.

31. S. P. Prakoso, S.-S. Sun, R. Saleh, Y.-T. Tao, C.-L. Wang, *ACS Appl. Mater. Interfaces* **2021**, *13*, 38365-38374.

32. R. Y. Zhang, N. X. Jin, T. Jia, L. Q. Wang, J. Liu, M. M. Nan, S. Qi, S. Q. Liu, Y. Y. Pan, *J. Mater. Chem. A* **2023**, *11*, 15380-15388.

33. Z. Chen, Y. Su, X. Tang, X. Zhang, C. Duan, F. Huang, Y. Li, *Sol. RRL* **2021**, *5*, 2100762.

34. W.-R. Cui, C.-R. Zhang, R.-P. Liang, J. Liu, J.-D. Qiu, *ACS Appl. Mater. Interfaces* **2021**, *13*, 31561-31568.

35. L. B. Zhang, B. Tang, J. B. Wu, R. Y. Li, P. Wang, *Adv. Mater* **2015**, *27*, 4889-4894.

36. Z. Wang, Y. T. Yan, X. P. Shen, C. D. Jin, Q. F. Sun, H. Q. Li, *J. Mater. Chem. A* **2019**, *7*, 20706-20712.

37. Q. Chen, Z. Pei, Y. Xu, Z. Li, Y. Yang, Y. Wei, Y. Ji, *Chem Sci* **2018**, *9*, 623-628.

38. Y. Zou, J. Zhao, J. Zhu, X. Guo, P. Chen, G. Duan, X. Liu, Y. Li, *ACS Appl. Mater. Interfaces* **2021**, *13*, 7617-7624.

39. X. Wu, G. Y. Chen, W. Zhang, X. Liu, H. Xu, *Adv. Sustain. Syst* **2017**, *1*, 1700046.

40. Y. Jin, J. Chang, Y. Shi, L. Shi, S. Hong, P. Wang, *J. Mater. Chem. A* **2018**, *6*, 7942-7949.

41. L. Zhu, T. Ding, M. Gao, C. K. N. Peh, G. W. Ho, *Adv. Energy Mater.* **2019**, *9*, 1900250.

42. X. Ma, W. Fang, Y. Guo, Z. Li, D. Chen, W. Ying, Z. Xu, C. Gao, X. Peng, *Small* **2019**, *15*, 1900354.

43. Z. Ai, Y. Zhao, R. Gao, L. Chen, T. Wen, W. Wang, T. Zhang, W. Ge, S. Song, *J. Clean. Prod* **2022**, *357*, 132000.

44. S. Ma, W. Qarony, M. I. Hossain, C. T. Yip, Y. H. Tsang, *Sol. Energy Mater. Sol. Cells* **2019**, *196*, 36-42.

45. X. Zhao, X. Ma, X. Peng, *Appl. Phys. A* **2019**, *125*, 537.

46. G. Wang, Y. Fu, A. Guo, T. Mei, J. Wang, J. Li, X. Wang, *Chem. Mater.* **2017**, *29*, 5629-5635.

47. W. Zhao, H. Gong, Y. Song, B. Li, N. Xu, X. Min, G. Liu, B. Zhu, L. Zhou, X.-X. Zhang, J. Zhu, *Adv. Funct. Mater.* **2021**, *31*, 2100025.

48. J. Q. Zhao, Y. W. Yang, C. H. Yang, Y. P. Tian, Y. Han, J. Liu, X. T. Yin, W. X. Que, *J. Mater. Chem. A* **2018**, *6*, 16196-16204.

49. H. Ghasemi, G. Ni, A. M. Marconnet, J. Loomis, S. Yerci, N. Miljkovic, G. Chen, *Nat. Commun.* **2014**, *5*, 4449.
